# Differential Effects of Peptidoglycan Recognition Proteins on Experimental Atopic and Contact Dermatitis Mediated by Treg and Th17 Cells

**DOI:** 10.1371/journal.pone.0024961

**Published:** 2011-09-16

**Authors:** Shin Yong Park, Dipika Gupta, Chang H. Kim, Roman Dziarski

**Affiliations:** 1 Indiana University School of Medicine–Northwest, Gary, Indiana, United States of America; 2 School of Veterinary Medicine, Purdue University, West Lafayette, Indiana, United States of America; Louisiana State University, United States of America

## Abstract

Skin protects the body from the environment and is an important component of the innate and adaptive immune systems. Atopic dermatitis and contact dermatitis are among the most frequent inflammatory skin diseases and are both determined by multigenic predisposition, environmental factors, and aberrant immune response. Peptidoglycan Recognition Proteins (Pglyrps) are expressed in the skin and we report here that they modulate sensitivity to experimentally-induced atopic dermatitis and contact dermatitis. *Pglyrp3* ^−/−^ and *Pglyrp4* ^−/−^ mice (but not *Pglyrp2* ^−/−^ mice) develop more severe oxazolone-induced atopic dermatitis than wild type (WT) mice. The common mechanism underlying this increased sensitivity of *Pglyrp3* ^−/−^ and *Pglyrp4* ^−/−^ mice to atopic dermatitis is reduced recruitment of Treg cells to the skin and enhanced production and activation Th17 cells in *Pglyrp3* ^−/−^ and *Pglyrp4* ^−/−^ mice, which results in more severe inflammation and keratinocyte proliferation. This mechanism is supported by decreased inflammation in *Pglyrp3* ^−/−^ mice following *in vivo* induction of Treg cells by vitamin D or after neutralization of IL-17. By contrast, *Pglyrp1* ^−/−^ mice develop less severe oxazolone-induced atopic dermatitis and also oxazolone-induced contact dermatitis than WT mice. Thus, Pglyrp3 and Pglyrp4 limit over-activation of Th17 cells by promoting accumulation of Treg cells at the site of chronic inflammation, which protects the skin from exaggerated inflammatory response to cell activators and allergens, whereas Pglyrp1 has an opposite pro-inflammatory effect in the skin.

## Introduction

Skin protects the body from the environment and is the largest organ in mammals. Besides forming a mechanical barrier, skin is an important component of the innate and adaptive immune systems rich in anti-microbial peptides and antigen-sensing cells, and it maintains the proper homeostatic balance between pro- and anti-inflammatory responses. Atopic dermatitis and contact dermatitis are among the most frequent inflammatory skin diseases, both determined by multigenic predisposition, environmental factors, and aberrant immune response. Atopic dermatitis has a prevalence of 15–30% in children and 2–10% in adults, involves loss of barrier function of the skin and type I hypersensitivity to environmental allergens, and is manifested by pruritic erythematosus skin eruptions and increased IgE response, often with aggravating bacterial infections [1]–[3]. Allergic contact dermatitis (contact hypersensitivity) has a prevalence of 2–20% and involves type IV (delayed-type) hypersensitivity to environmental allergens, manifested by erythematosus skin infiltrations with inflammatory cells [2], [4], [5].

Peptidoglycan Recognition Proteins (PGRPs or Pglyrps) are a family of innate immunity proteins expressed in the skin. PGRPs are conserved from insects to mammals, recognize bacterial peptidoglycan, and function in antibacterial immunity. Mammals have four PGRPs, Pglyrp1, Pglyrp2, Pglyrp3, and Pglyrp4, which were initially named PGRP-S, PGRP-L, PGRP-Iα, and PGRP-Iβ, respectively [6], [7]. Three PGRPs, Pglyrp1, Pglyrp3, and Pglyrp4 are directly bactericidal [8]–[11], whereas Pglyrp2 is an N-acetylmuramoyl-L-alanine amidase that hydrolyzes peptidoglycan [12], [13]. Pglyrp1 is highly expressed in PMN's granules and to a much lower extent in other cells [7], [14], [15]. Pglyrp2 is constitutively expressed in the liver and secreted into blood, and its expression is induced in keratinocytes and other epithelial cells [12], [13], [16]–[20]. Pglyrp3 and Pglyrp4 have the highest expression in the skin and are also expressed in the salivary glands, throat, tongue, esophagus, stomach, intestine, and eyes [8], [21], [22]. Similar to Pglyrp1 and Pglyrp2, Pglyrp3 and Pglyrp4 are secreted and their expression in these tissues is both constitutive and inducible.

We hypothesized that PGRPs play a role in the development atopic dermatitis and contact dermatitis because of (a) the prominent expression of PGRPs in the skin, (b) the location of *Pglyrp3* and *Pglyrp4* genes in the epidermal differentiation gene cluster in the psoriasis sensitivity *psors4* locus, (c) coordinated expression of *Pglyrp3* and *Pglyrp4* with other genes in the *psors4* locus, (d) previous evidence of genetic association of *Pglyrp3* and *Pglyrp4* variants with psoriasis, which is another genetically- and environmentally-determined skin disease [23], [24], and (e) the ability of mammalian PGRPs to protect mice against experimental colitis [22] and to modulate the development of experimental arthritis [25]. Here we tested this hypothesis using PGRP-deficient mice and mouse models of chemically-induced atopic dermatitis and contact dermatitis.

Our results show that *Pglyrp3* ^−/−^ and *Pglyrp4* ^−/−^ mice (but not *Pglyrp2* ^−/−^ mice) are more sensitive to the development of experimental atopic dermatitis than wild type (WT) mice. The common mechanism underlying this increased sensitivity of *Pglyrp3* ^−/−^ and *Pglyrp4* ^−/−^ mice is reduced recruitment of Treg cells to the skin and enhanced production and activation Th17 cells in *Pglyrp3* ^−/−^ and *Pglyrp4* ^−/−^ mice, which results in more severe inflammation and keratinocyte proliferation. By contrast, *Pglyrp1* ^−/−^ mice are less sensitive than WT mice to both experimental atopic dermatitis and contact dermatitis. Thus, Pglyrp3 and Pglyrp4 limit over-activation of Th17 cells by promoting accumulation of Treg cells at the site of chronic inflammation, which protects the skin from exaggerated inflammatory response to cell activators and allergens. By contrast, Pglyrp1 has an opposite pro-inflammatory effect in the skin.

## Results

### *Pglyrp3* ^−/−^ and *Pglyrp4* ^−/−^ mice have enhanced inflammatory response in the oxazolone model of atopic dermatitis

**Figure 1:**
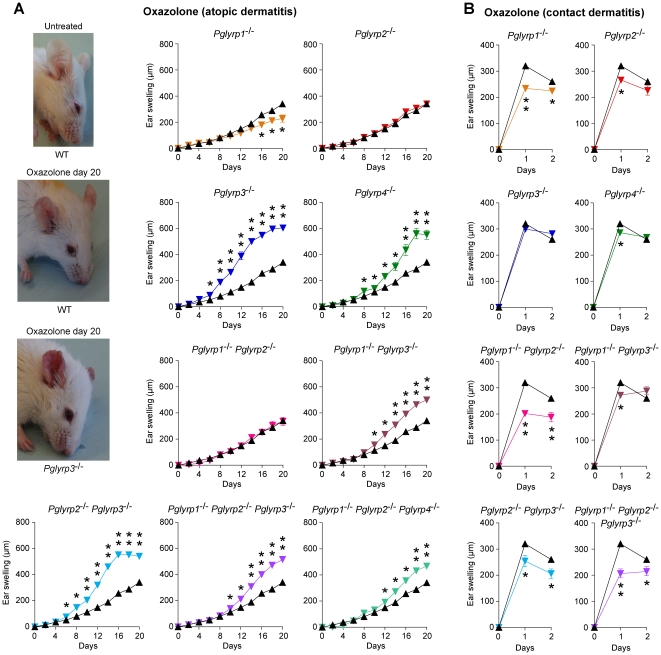
*Pglyrp3* ^−/−^ and *Pglyrp4* ^−/−^ mice have enhanced response in the oxazolone model of atopic dermatitis and *Pglyrp1* ^−/−^ and other *Pglyrp* ^−/−^ mice have reduced response in the oxazolone model of contact dermatitis. (**A**) Atopic dermatitis model: sensitization followed by 10 applications of oxazolone to the ears every other day induces mild inflammation (left) and mild ear swelling (right) in WT mice (black triangles) and severe inflammation with increased redness, scaling, and extensive ear swelling in *Pglyrp3* ^−/−^ and *Pglyrp4* ^−/−^ mice (color triangles). (**B**) Contact dermatitis model: sensitization followed by a single application of oxazolone to the ears induces strong ear swelling in WT mice (black triangles) and reduced ear swelling in *Pglyrp1* ^−/−^, *Pglyrp2* ^−/−^, and *Pglyrp4* ^−/−^ mice (color triangles). Means ± SEM (SEM were often smaller than the symbols in this and other figures); N = 9–17 mice/group; significance of differences between *Pglyrp* ^−/−^ and WT mice: *, P<0.02; **, P<0.0001.

Repeated epicutaneous sensitization with oxazolone is an established mouse model of atopic dermatitis [3], [26]. Initial sensitization of mice with oxazolone through abdominal skin, followed by 6-day rest and application of oxazolone to the ears every other day for 20 days induced in WT BALB/c mice progressive moderate inflammation manifested by some redness and swelling (Figure 1A). Similar application of oxazolone to *Pglyrp3* ^−/−^ or *Pglyrp4* ^−/−^ mice induced significantly enhanced inflammation, manifested first by increased redness and significantly increased swelling, accompanied by scaling (Figure 1A). This enhanced response was unique for *Pglyrp3* ^−/−^ and *Pglyrp4* ^−/−^ mice, because it was not observed in *Pglyrp1* ^−/−^, *Pglyrp2* ^−/−^, and *Pglyrp1* ^−/−^ *Pglyrp2* ^−/−^ mice, and was dominant, because it was still observed in *Pglyrp1* ^−/−^ *Pglyrp3* ^−/−^ and *Pglyrp2* ^−/−^ *Pglyrp3* ^−/−^ double-knockout mice and in *Pglyrp1* ^−/−^ *Pglyrp2* ^−/−^ *Pglyrp3* ^−/−^ and *Pglyrp1* ^−/−^ *Pglyrp2* ^−/−^ *Pglyrp4* ^−/−^ triple-knockout mice (Figure 1A). Deletion of *Pglyrp1* had the opposite effect – *Pglyrp1* ^−/−^ single knockout mice showed reduced ear swelling on days 16 through 20 of oxazolone application (Figure 1A). These results indicate that in WT mice both Pglyrp3 and Pglyrp4 have a protective effect against severe atopic dermatitis-like inflammation, whereas Pglyrp1 has an enhancing proinflammatory effect and Pglyrp2 has little effect on the response to oxazolone.

**Figure 2:**
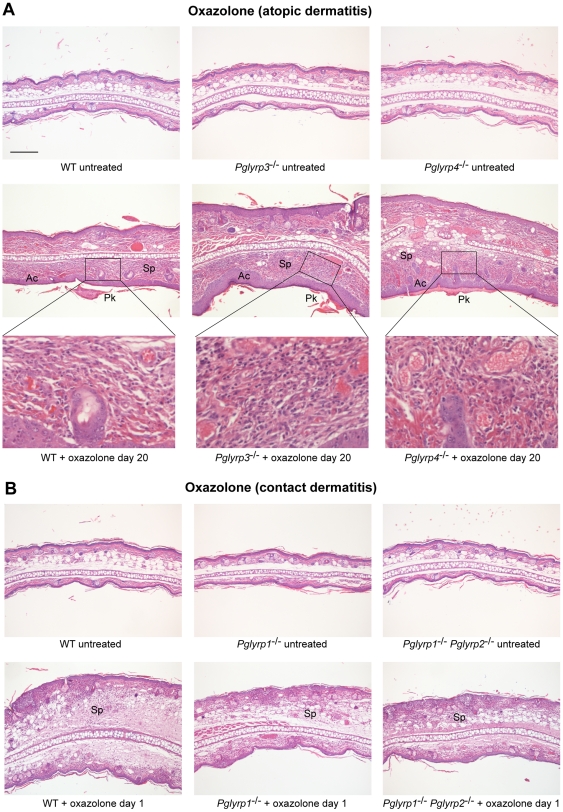
Ear histology in WT and *Pglyrp*-deficient mice in atopic dermatitis and contact dermatitis models of skin inflammation. (**A**) Oxazolone model of atopic dermatitis: sensitization and 10 applications of oxazolone to the ears every other day induced acanthosis (Ac), parakeratosis (Pk), marked thickening of the sub-epidermal layer with spongiosis (Sp) and dense cellular infiltrates of primarily mononuclear and some polymorphonuclear cells (high magnification insets), that were all highly prominent in *Pglyrp3* ^−/−^ mice and *Pglyrp4* ^−/−^ mice and much less severe in WT mice. (**B**) Oxazolone model of contact dermatitis: sensitization and a single application of oxazolone to the ears induced strong inflammatory response in WT mice with marked spongiosis of the sub-epidermal layer (Sp) and cellular infiltrates of epidermal and sub-epidermal layers, composed of mononuclear and polymorphonuclear cells; *Pglyrp1* ^−/−^ and *Pglyrp1* ^−/−^ *Pglyrp2* ^−/−^ mice still had cellular infiltrates, but had substantially reduced swelling, compared to WT mice, mostly due to reduced edema. H&E stained cross-sections; bar = 200 µm for all panels, except high magnification insets (the magnified areas are shown by rectangles).

To determine the pathologic basis of higher sensitivity of *Pglyrp3* ^−/−^ and *Pglyrp4* ^−/−^ mice to oxazolone, we compared the histology of oxazolone-induced skin lesions in WT and *Pglyrp*-deficient mice. Ears in untreated WT mice have one- to two-cell thick epidermis and few-cell thick subepidermal layer with blood vessels, sebaceous glands, hair follicles, muscle bundles, and central fat and connective tissue layer, with total thickness of approximately 200 µm. Histology of all untreated *Pglyrp*-deficient mice was similar to WT mice (Figure 2). Sensitization and 10 oxazolone applications to the ears (every other day) induced strong inflammatory response that was very severe in *Pglyrp3* ^−/−^ mice and *Pglyrp4* ^−/−^ mice. Cross-sections of the oxazolone-treated ears revealed severe acanthosis (thickening of the epidermis due to proliferation of keratinocytes), parakeratosis (retention of keratinocytes' nuclei in stratum corneum), and marked thickening of the sub-epidermal layer with spongiosis (intercellular edema) and dense cellular infiltrates (composed primarily of mononuclear cells and some polymorphonuclear cells) that were all highly prominent in *Pglyrp3* ^−/−^ mice and *Pglyrp4* ^−/−^ mice (Figure 2A) and in all other double and triple knockout mice deficient in *Pglyrp3* (not shown). All these changes are highly characteristic of atopic dermatitis lesions. These mice did not develop rete pegs (downward papillary projections of epidermis), which are characteristic of psoriasis, but not atopic dermatitis. WT mice (Figure 2A), *Pglyrp1* ^−/−^ mice, *Pglyrp2* ^−/−^ mice, and *Pglyrp1* ^−/−^ *Pglyrp2* ^−/−^ mice (not shown) all showed much less severe acanthosis, parakeratosis, edema, and cell infiltrations, and less thickening of the subepidermal layer.

**Figure 3:**
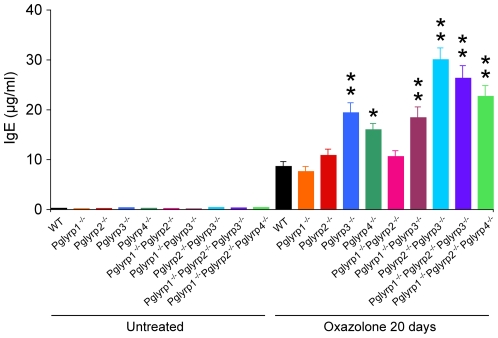
*Pglyrp3* ^−/−^ and *Pglyrp4* ^−/−^ mice have higher serum IgE levels than WT mice in the oxazolone model of atopic dermatitis. Sensitization followed by 10 applications of oxazolone to the ears every other day induced higher level of serum IgE in *Pglyrp3* ^−/−^ and *Pglyrp4* ^−/−^ mice than in WT, *Pglyrp1* ^−/−^ or *Pglyrp2* ^−/−^ mice; means ± SEM of 8–20 mice/group; *, P<0.002; **, P<0.0001, *Pglyrp* ^−/−^ versus WT.

Inflammatory response to oxazolone was accompanied by a marked increase in the concentration of serum IgE, consistent with oxazolone inflammation being a model of atopic dermatitis (which is type I hypersensitivity associated with increased IgE production). The serum IgE levels in *Pglyrp3* ^−/−^ mice and *Pglyrp4* ^−/−^ mice, as well as *Pglyrp1* ^−/−^ *Pglyrp3* ^−/−^ and *Pglyrp2* ^−/−^ *Pglyrp3* ^−/−^ double-knockout mice and in *Pglyrp1* ^−/−^ *Pglyrp2* ^−/−^ *Pglyrp3* ^−/−^ and *Pglyrp1* ^−/−^ *Pglyrp2* ^−/−^ *Pglyrp4* ^−/−^ triple-knockout mice were all significantly higher than in WT and in *Pglyrp1* ^−/−^, *Pglyrp2* ^−/−^, and *Pglyrp1* ^−/−^ *Pglyrp2* ^−/−^ mice (Figure 3). Pglyrp2 also played a minor role, because *Pglyrp2* ^−/−^ *Pglyrp3* ^−/−^ double-knockout mice and *Pglyrp1* ^−/−^ *Pglyrp2* ^−/−^ *Pglyrp3* ^−/−^ and *Pglyrp1* ^−/−^ *Pglyrp2* ^−/−^ *Pglyrp4* ^−/−^ triple-knockout mice had higher serum IgE levels than *Pglyrp3* ^−/−^ and *Pglyrp4* ^−/−^ single-knockout mice.

These results demonstrate that deletion of *Pglyrp3* or *Pglyrp4* highly predisposes mice to atopic dermatitis-like lesions in response to oxazolone, and thus in WT mice Pglyrp3 or Pglyrp4 protect the skin from excessive inflammation in the oxazolone model of atopic dermatitis.

### *Pglyrp*-deficient mice have reduced response in the oxazolone model of contact dermatitis

Single epicutaneous sensitization with oxazolone followed by a single epicutaneous challenge in a different area is an established mouse model of allergic contact dermatitis (contact hypersensitivity) due to type IV hypersensitivity [27], [28]. Sensitization of mice with oxazolone through abdominal skin, followed by 6-day rest and a single application of oxazolone to the ears induced on the next day a strong inflammatory response in WT BALB/c mice, manifested by redness and swelling (Figure 1B). Similar application of oxazolone to *Pglyrp*-deficient mice induced significantly lower inflammation, manifested by significantly reduced ear swelling compared to WT mice, especially in *Pglyrp1* ^−/−^ mice, and to some extent in *Pglyrp2* ^−/−^ and *Pglyrp4* ^−/−^ mice. The reduced responsiveness was dominant, because it was still observed in *Pglyrp1* ^−/−^ *Pglyrp2* ^−/−^ (most affected), *Pglyrp1* ^−/−^ *Pglyrp3* ^−/−^, and *Pglyrp2* ^−/−^ *Pglyrp3* ^−/−^ double-knockout mice and in *Pglyrp1* ^−/−^ *Pglyrp2* ^−/−^ *Pglyrp3* ^−/−^ triple-knockout mice (Figure 1B).

To determine the pathologic basis of this lower response to oxazolone in the contact dermatitis model in *Pglyrp*-deficient mice, we compared the ear histology in oxazolone-treated WT and *Pglyrp*-deficient mice. Sensitization and single oxazolone application to the ears induced strong inflammatory response in WT mice manifested by marked spongiosis of the sub-epidermal layer with cellular infiltrates of epidermal and sub-epidermal layers with mononuclear and polymorphonuclear cells (but no acanthosis, parakeratosis, rete pegs, or scaling, which histologically differentiates contact dermatitis from atopic dermatitis and psoriasis) (Figure 2B). *Pglyrp1* ^−/−^ and *Pglyrp1* ^−/−^ *Pglyrp2* ^−/−^ mice still had cellular infiltrates, but had substantially reduced swelling, compared to WT mice, mostly due to reduced edema (Figure 2B). These results indicate that in WT mice Pglyrp1 (and also to some extent Pglyrp2 and Pglyrp4) has an enhancing proinflammatory role in this model of contact dermatitis.

Thus, altogether our results indicate that individual PGRPs have selective and distinct effects on skin inflammation. In WT mice Pglyrp3 and Pglyrp4 offer protection in the oxazolone atopic dermatitis model, Pglyrp1 has a pro-inflammatory effect both atopic and contact dermatitis models, and Pglyrp2 has little effect on the atopic dermatitis and has a pro-inflammatory effect in the contact dermatitis model of skin inflammation.

### Expression of PGRPs in inflamed ears

**Figure 4:**
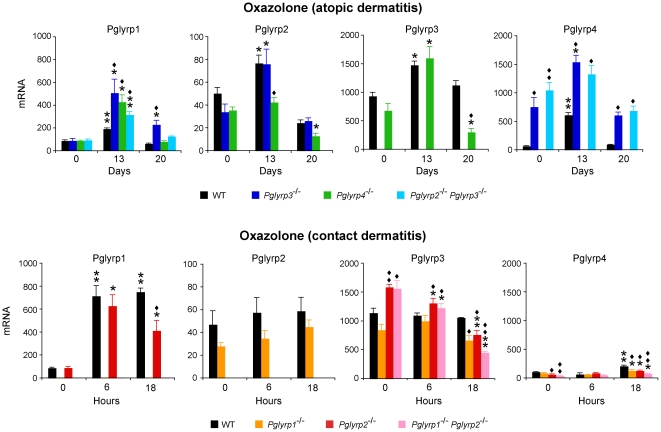
Pglyrp1, Pglyrp2, Pglyrp3, and Pglyrp4 expression is increased in the affected skin in the atopic dermatitis and contact dermatitis models of inflammation. The amounts of each PGRP mRNA in WT mice and in the indicated *Pglyrp*-deficient mice sensitized and treated with oxazolone every other day (atopic dermatitis model) or sensitized and treated with oxazolone at 0 hrs only (contact dermatitis model) were measured by qRT-PCR. The results are means of 3–4 mice ± SEM; *, P<0.04; **, P<0.001, treated versus untreated; ♦, P<0.04; ♦♦, P<0.001, *Pglyrp* ^−/−^ versus WT.

To gain further insight how individual PGRPs influence sensitivity to both models of skin inflammation, we compared expression of all PGRPs in the ears in untreated and oxazolone-treated mice. In both atopic and contact dermatitis models treatment with oxazolone induced increased Pglyrp1 mRNA expression in the ears in all strains of mice (except *Pglyrp1* ^−/−^ mice, in which *Pglyrp1* gene is deleted) that was significantly higher than in untreated mice (Figure 4). The expression of Pglyrp1 was significantly higher in all *Pglyrp*-deficient mice^−^ than in WT mice early (day 13) in the atopic dermatitis model, which correlates with their higher inflammatory response. This increase in Pglyrp1 mRNA in the ears is likely due to increased infiltration with PMNs, because PMNs highly express Pglyrp1 and expression of Pglyrp1 is not inducible in any of the cell types previously studied, including lymphocytes, monocytes, macrophages, and keratinocytes [8], [14], [15]. The expression of Pglyrp2 was only modestly increased early in the atopic dermatitis model and returned to the untreated level later in this model, and was not significantly changed at any time point in the contact dermatitis model, consistent with the little effect of Pglyrp2 in both atopic and contact dermatitis models (Figure 1). Pglyrp1 and Pglyrp2 were also constitutively expressed in the cervical lymph nodes at a similar level as in the ears, but following oxazolone treatment their expression in the cervical lymph nodes did not significantly change (data not shown), despite extensive stimulation and expansion of immune cells in the draining lymph nodes, which increased in diameter from <0.5 mm in untreated mice to >3 mm after 20 days of oxazolone treatment.

**Figure 5:**
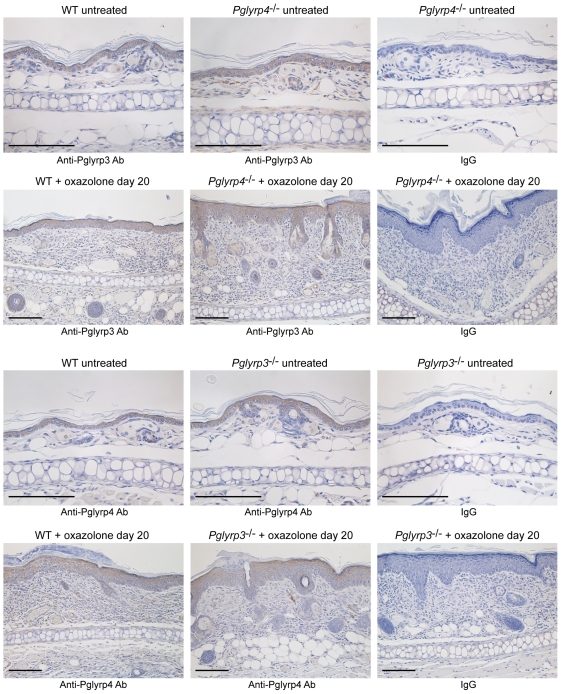
Pglyrp3 and Pglyrp4 are expressed primarily in the differentiated epidermal cells in both untreated and oxazolone-treated skin in the atopic dermatitis model. Transverse sections of the ears from either untreated or oxazolone-treated mice (sensitization followed by 10 applications of oxazolone to the ears every other day) were stained by an immunoperoxidase method with either anti-Pglyrp3 or anti-Pglyrp4 antibodies or IgG (negative control); bar = 50 µm.

Pglyrp3 has high constitutive expression in untreated skin [8], [21]. Pglyrp3 mRNA expression initially increased and later decreased in the atopic dermatitis model, and decreased in the contact dermatitis model. Pglyrp4 has lower constitutive expression in untreated skin than Pglyrp3. Expression of Pglyrp4 mRNA was highly increased in the atopic dermatitis model and was the highest in *Pglyrp2* ^−/−^ *Pglyrp3* ^−/−^ and *Pglyrp3* ^−/−^ mice, suggesting a possibility of compensatory expression of Pglyrp4 in mice deficient in Pglyrp3 or changes in the regulation of transcription or stability of Pglyrp4 mRNA, since Pglyrp3 and Pglyrp4 genes are tightly linked in the psoriasis sensitivity locus on chromosome 3 in mice and their expression is correlated with the expression of keratinocytes differentiation genes [21]. To further investigate which cells express Pglyrp3 and Pglyrp4 in the untreated and oxazolone-treated skin, we analyzed the expression of Pglyrp3 and Pglyrp4 proteins by immunohistochemistry. Pglyrp3 and Pglyrp4 were expressed in epidermal keratinocytes in untreated ears, and in oxazolone-treated ears they were primarily expressed in the upper layers of differentiated keratinocytes (Figure 5). Pglyrp3 and Pglyrp4 expression was not induced in other cell types, such as infiltrating inflammatory cells in oxazolone-treated ears. Moreover, Pglyrp3 and Pglyrp4 were not expressed in the cervical lymph nodes in untreated mice and their expression there was not induced following oxazolone treatment (data not shown). These results indicate that Pglyrp3 and Pglyrp4 are primarily expressed in keratinocytes, but not in immune or inflammatory cells, and thus these results raise the possibility that Pglyrp3 and Pglyrp4 exert their protective effects in the oxazolone-induced atopic dermatitis by modulating the function of keratinocytes, which are known to produce many pro-inflammatory cytokines and chemokines.

### *Pglyrp3* ^−/−^ and *Pglyrp4* ^−/−^ mice have increased Th17 cells and Th17 responses in the skin

To determine the cellular basis for the differences in the inflammatory responses in *Pglyrp*-deficient mice, we next determined the types of inflammatory cells in the ears in both models of skin inflammation, and then we determined which cell types significantly differed in *Pglyrp*-deficient mice compared to WT mice. This was first accomplished by measuring the amounts of mRNA for several marker genes characteristic of various immune and inflammatory cell types in the untreated and the affected ears. To determine which marker genes (and thus cell types) are increased or decreased in *Pglyrp*-deficient mice compared to WT mice, we calculated how many times higher or lower they were induced in *Pglyrp*-deficient mice than in WT mice (fold induction in *Pglyrp*-deficient mice/fold induction in WT mice).

**Figure 6:**
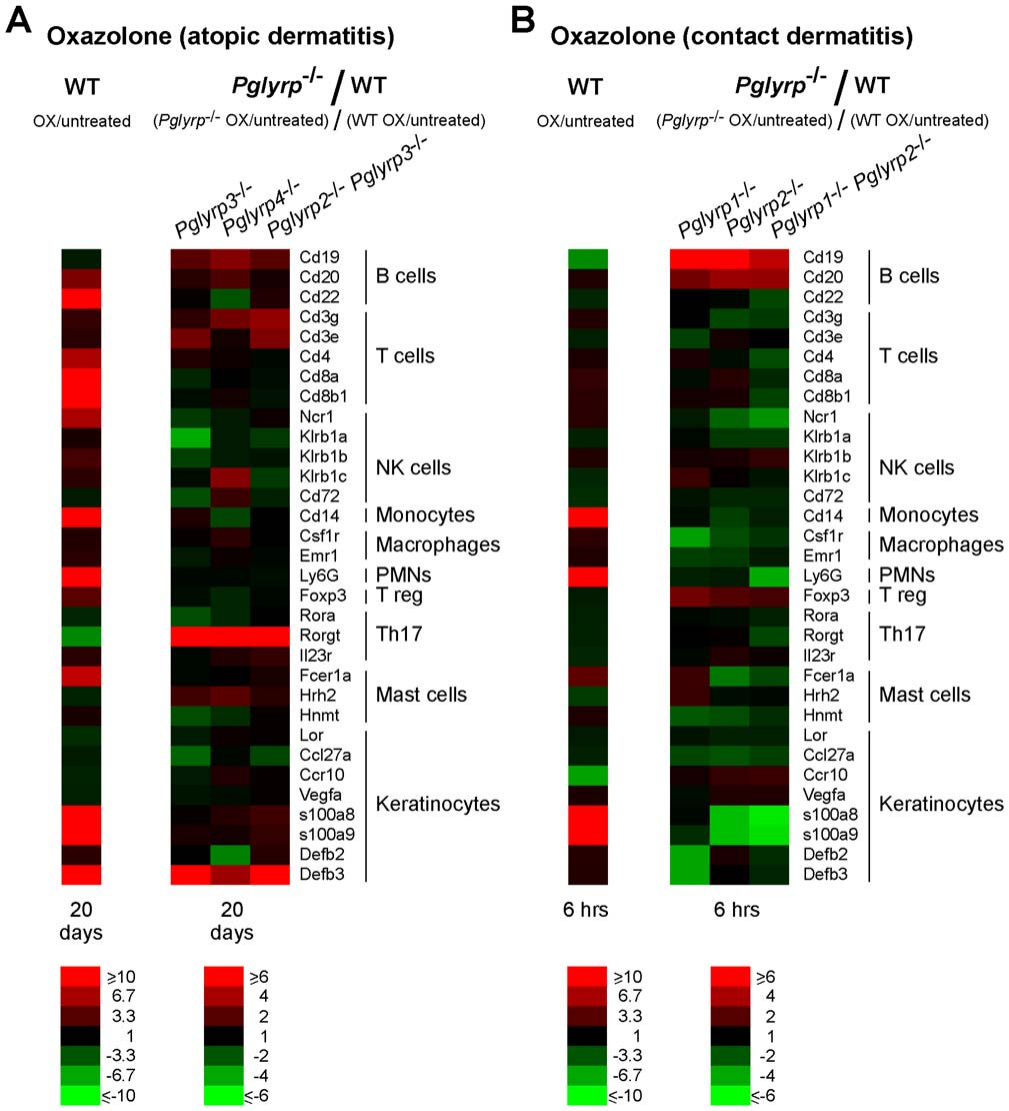
*Pglyrp3* ^−/−^ and *Pglyrp4* ^−/−^ mice have increased Th17 cells in the affected skin in the atopic dermatitis model, whereas *Pglyrp1* ^−/−^ and *Pglyrp2* ^−/−^ mice have most cell types decreased (except B cells and Treg cells) in the contact dermatitis model. Expression of a panel of marker genes characteristic of various inflammatory cell types in the ears of mice after (**A**) sensitization and 10 applications of oxazolone to the ears every other day, or (**B**) after sensitization and 6 hrs after a single application of oxazolone to the ears, measured by qRT-PCR is shown. For WT mice (left panels in A and B), the ratio of the amount of mRNA in oxazolone-treated to untreated mice for each gene (fold induction by oxazolone) is shown; for *Pglyrp* ^−/−^ mice (right panels in A and B), the results are the ratios of fold induction of each gene by oxazolone in *Pglyrp* ^−/−^ mice to fold induction of each gene by oxazolone in WT mice (which represents the fold difference in the response to oxazolone in *Pglyrp* ^−/−^ versus WT mice). The results are means of 3 arrays from 4–5 mice/group in heat map format. The means ± SEM bar graphs for these results are shown in Figures S1 and S2.

Prolonged treatment with oxazolone in the atopic dermatitis model induced increases in mRNA of several cell types in WT mice, and especially mature B cells, CD8^+^ T cells, monocytes, PMNs, and mast cells, as well as changes associated with de-differentiation, proliferation, and activation of keratinocytes, which are all expected changes consistent with the atopic dermatitis model (Figure 6A and Figure S1). Atopic dermatitis-sensitive mice (*Pglyrp3* ^−/−^, *Pglyrp4* ^−/−^, and *Pglyrp2* ^−/−^ *Pglyrp3* ^−/−^ mice) had increased expression of genes characteristic of B cells and T cells. Also increased was Rorγt mRNA, which is preferentially expressed in Th17 cells [29] (Figure 6A and Figure S1).

Short-term treatment with oxazolone in the contact dermatitis model in WT mice induced increases primarily in monocytes and PMNs at 6 hrs after challenge (Figure 6B and Figure S2) and later in T cells (18 hrs, not shown). Contact dermatitis-resistant mice (*Pglyrp1* ^−/−^, *Pglyrp2* ^−/−^, and *Pglyrp1* ^−/−^ *Pglyrp2* ^−/−^ mice) had initially (6 hrs after challenge) increased B cells and Treg cells and decreased other cell-types, compared to WT mice (Figure 6B and Figure S2). Later (18 hrs after challenge) the cell markers in *Pglyrp2* ^−/−^ and *Pglyrp1* ^−/−^ *Pglyrp2* ^−/−^ mice were similar (most cells) or increased (monocytes), compared to WT mice (not shown), consistent with comparable cell infiltrates seen in all mice on tissue sections on day 1 (Figure 2B).

To further define the cell types responsible for differential sensitivity of *Pglyrp*-deficient mice to atopic dermatitis and contact dermatitis, we measured the expression of an extended panel of cytokines, chemokines, and other marker genes characteristic of Th1, Th2, Th17, Treg, NK, and other cell types to determine which of these genes were differentially induced in the affected skin in *Pglyrp*-deficient mice, compared to WT mice. We included these cell types, because in addition to Th1 and Th2 cells, Th17 cells and other cell types may also be involved in these skin diseases.

**Figure 7:**
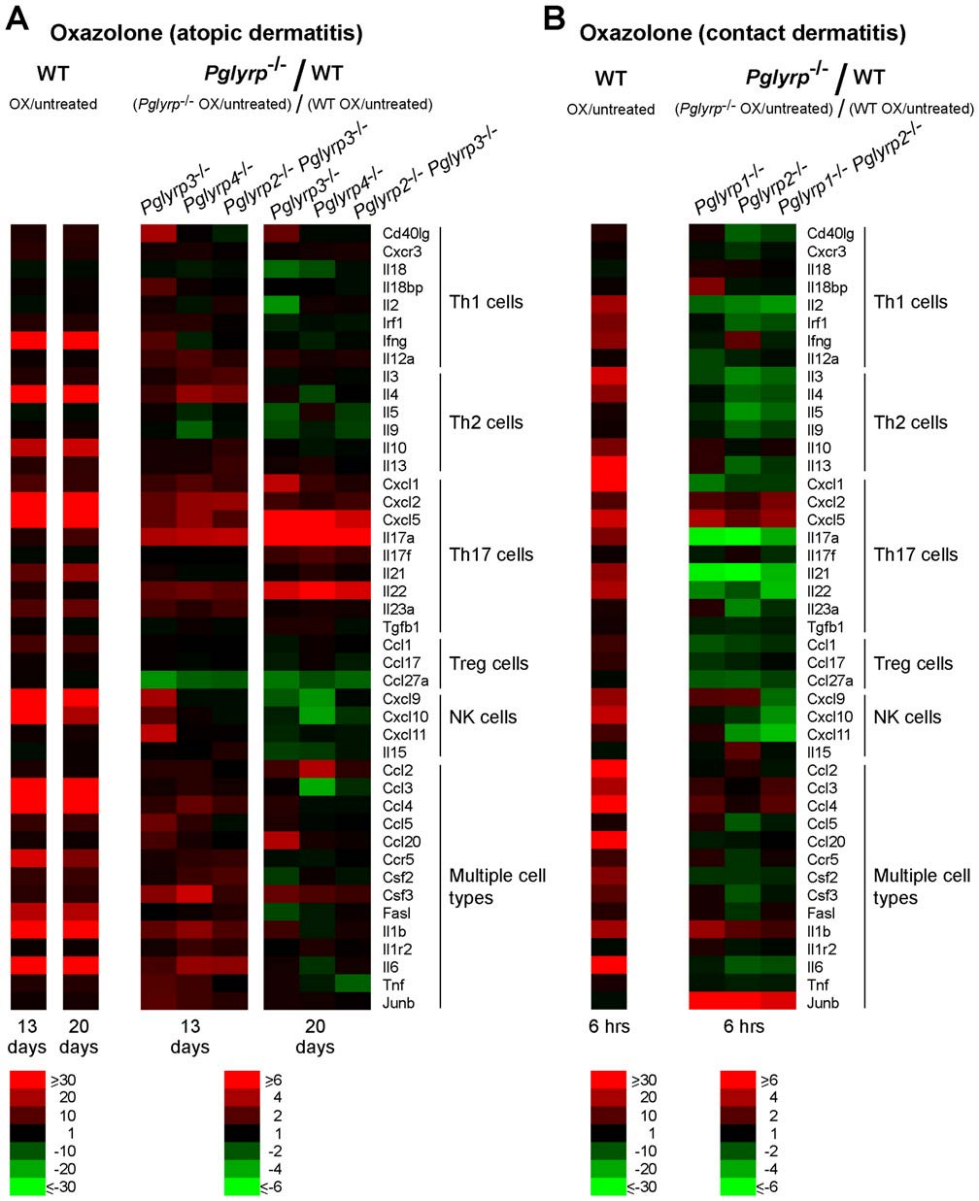
Th17 gene expression profile is preferentially induced in the atopic dermatitis model in *Pglyrp3* ^−/−^ and *Pglyrp4* ^−/−^ mice, whereas expression of most immune genes is reduced in the contact dermatitis model in *Pglyrp* ^−/−^ mice. Expression of a panel of cytokines, chemokines, and other marker genes characteristic of Th1, Th2, Th17, Treg, NK, and other cell types in the ears of mice was measured by qRT-PCR. (**A**) After sensitization and 10 applications of oxazolone to the ears every other day the expression of several Th17 marker genes was higher in *Pglyrp3* ^−/−^ and *Pglyrp4* ^−/−^ mice than in WT mice. (**B**) After sensitization and a single application of oxazolone to the ears the expression of most of immune marker genes was lower in *Pglyrp* ^−/−^ mice than in WT mice. For WT mice (left panels in A and B), the ratio of the amount of mRNA in oxazolone-treated to untreated mice for each gene (fold induction by oxazolone) is shown; for *Pglyrp* ^−/−^ mice (right panels in A and B), the results are the ratios of fold induction of each gene by oxazolone in *Pglyrp* ^−/−^ mice to fold induction of each gene by oxazolone in WT mice (which represents the fold difference in the response to oxazolone in *Pglyrp* ^−/−^ versus WT mice). The results are means of 3 arrays from 4–5 mice/group in heat map format. The means ± SEM bar graphs for these results are shown in Figures S3, S4, and S5.

Prolonged epicutaneous sensitization with oxazolone (20 days), which induced atopic dermatitis-like skin inflammation, was accompanied in WT mice by high activation (more than 15-fold) of 14 out of 44 studied genes in the affected skin. These genes were Ifng (Th1), Il4 and Il10 (Th2), Cxcl2, Cxcl5, and Il21 (Th17), Cxcl9 and Cxcl10 (NK cells), and Ccl3, Ccl4, Ccr5, Fasl, Il1b, and Il6 (several cell types) (Figure 7A and Figures S3 and S4). Atopic dermatitis-sensitive mice initially (day 13) had several genes activated higher than in WT mice, characteristic of several cell types, including Th2 and Th17 (Figure 7A and Figure S3). Later (day 20), atopic dermatitis-sensitive mice (*Pglyrp3* ^−/−^, *Pglyrp4* ^−/−^, and *Pglyrp2* ^−/−^ *Pglyrp3* ^−/−^ mice) had four genes characteristic of Th17 cells (Cxcl1, Cxcl5, IL17a, IL22) and one gene characteristic of several cell types (Ccl2) activated more than three-fold higher in *Pglyrp*-deficient than in WT mice (Figure 7A and Figure S4).

A single oxazolone challenge in sensitized WT mice (in the contact dermatitis model) also induced many genes characteristic of several cell types, and the early (6 hrs) activation of these genes in *Pglyrp*-deficient mice was mostly reduced, compared to WT mice (Figure 7B and Figure S5). These results are consistent with lower clinical responses of *Pglyrp*-deficient mice to a single oxazolone challenge in the contact dermatitis model (Figures 1B and 2B).

The above results indicate that the atopic dermatitis-sensitive *Pglyrp*-deficient mice have increased activity of Th17 cells in the affected skin, compared to WT mice. To further investigate the role Th17 cells (and other Th cell types) in increased sensitivity of *Pglyrp*-deficient mice in atopic dermatitis model, we used flow cytometry to directly measure Th cell types in the ears, draining lymph nodes, and the spleen.

**Figure 8:**
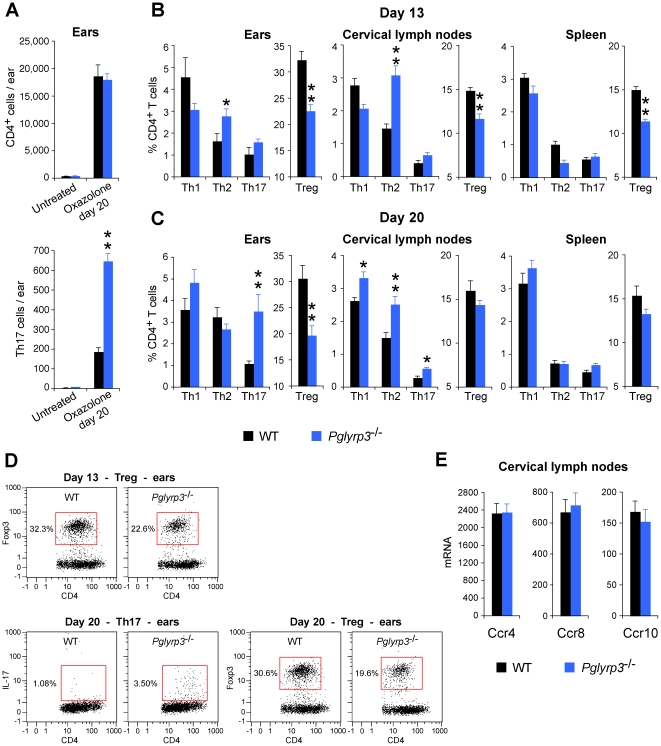
*Pglyrp3* ^−/−^ mice have high numbers Th17 and low numbers of Treg cells in the affected skin in the oxazolone model of atopic dermatitis. (**A**) Numbers of CD4^+^ cells and Th17 cells in the ears or (**B–D**) percentages of Th1, Th2, Th17, and Treg cells in the ears, cervical lymph nodes, and spleen in sensitized WT and *Pglyrp3* ^−/−^ mice on days 13 or 20 of ear treatment with oxazolone, measured by flow cytometry; means ± SEM of 5–9 mice/group (*, P<0.05; **, P<0.005; *Pglyrp3* ^−/−^ versus WT) or representative dot plots are shown. (**E**) Expression of receptors for chemokines that attract Treg cells in cervical lymph nodes of sensitized WT and *Pglyrp3* ^−/−^ mice on day 20 of ear treatment with oxazolone measured by qRT-PCR; amounts of mRNA are shown as means ± SEM of 3 arrays from 5 mice/group.

Untreated ears in WT and *Pglyrp* ^−/−^ mice had <400 CD4^+^ cells/ear, whereas after sensitization and 20 days of oxazolone treatment the numbers of CD4^+^ cells/ear increased >50 times to ∼18,000–19,000/ear in WT and *Pglyrp3* ^−/−^ mice (Figure 8A). Regarding Th cell subpopulations, oxazolone treatment for 13 days induced significantly higher numbers of Th2 cells (CD4^+^IL-4^+^) in the affected ears in *Pglyrp3* ^−/−^ mice compared to WT mice, whereas oxazolone treatment for 20 days induced significantly higher numbers of Th17 cells (CD4^+^IL-17^+^) in the affected ears in *Pglyrp3* ^−/−^ mice compared to WT mice (Figure 8A, B and C). Thus, on day 20 in *Pglyrp3* ^−/−^ mice the numbers of Th17 cells in the ears increased from undetectable (<10/ear) to ∼650 Th17 cells/ear, 3.5 times higher than in WT mice (Figure 8A). Virtually all detectable IL-17^+^ cells in the oxazolone-treated ears were CD4^+^ (Th17 cells) and there were very few (<50/ear, not shown) other IL-17^+^ cells in the inflamed skin (such as CD8^+^, γ/δ T cells, or NKT cells), and therefore the observed increases in the numbers IL-17^+^ cells mostly represent increases in Th17 cells (CD4^+^IL-17^+^). There was no significant difference in the numbers of Th1 (CD4^+^IFN-γ^+^) and Th2 (CD4^+^IL-4^+^) cells in the ears of WT and *Pglyrp3* ^−/−^ mice on day 20 (Figure 8C). Oxazolone-treated mice had substantially swollen cervical lymph nodes (>3 mm in diameter, compared to <0.5 mm in untreated mice), where on day 13 the numbers of Th2 cells and on day 20 the numbers of all Th cell types were significantly higher in *Pglyrp3* ^−/−^ mice compared to WT mice (Figure 8B and C).

These results indicate initial (day 13) preferential activation of Th2 cells in the affected ears and draining lymph nodes in *Pglyrp3* ^−/−^ mice compared to WT mice, consistent with B-cell-dependence of atopic dermatitis model. However, continued treatment with oxazolone (20 days) showed a switch to preferential infiltration of the affected ears with Th17 cells in *Pglyrp3* ^−/−^ mice compared to WT mice (Figure 8), consistent with our mRNA gene expression data (Figures 6 and 7 and Figures S1, S3, and S4).

### IL-17 is required for enhanced response to oxazolone in *Pglyrp3* ^−/−^ mice

**Figure 9:**
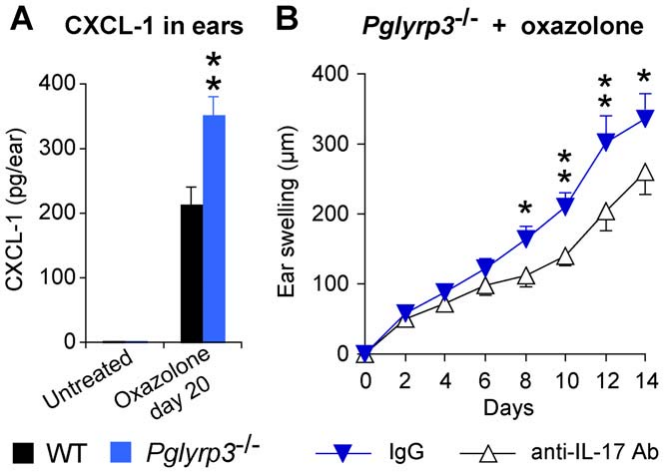
IL-17 is required for enhanced response to oxazolone in *Pglyrp3* ^−/−^ mice. (**A**) The level of IL-17-induced chemokine, CXCL-1, is higher in the ears of *Pglyrp3* ^−/−^ mice than WT mice after sensitization and application of oxazolone for 20 days. (**B**) Ear swelling in *Pglyrp3* ^−/−^ mice sensitized and treated 7 times with oxazolone every other day and also treated with neutralizing anti-IL-17 mAbs is lower than in *Pglyrp3* ^−/−^mice similarly treated with oxazolone and isotype control IgG. Means ± SEM; N = 6 mice/group; significance of differences between *Pglyrp3* ^−/−^ and WT mice (A) or IgG control and anti-IL-17 mAbs-treated mice (B): *, P<0.05; **, P<0.005.

To further study the role of IL-17 (Th17 cytokine) in high sensitivity of *Pglyrp3* ^−/−^ mice to oxazolone-induced atopic dermatitis, we determined the protein levels of an IL-17-induced chemokine, CXCL-1, in the ears of WT and *Pglyrp3* ^−/−^ mice. CXCL-1 was undetectable (<7 pg/ear) in the ears of untreated mice, and after sensitization and 20 days of skin treatment with oxazolone, the amount of CXCL-1 increased to >350 pg/ear in *Pglyrp3* ^−/−^ mice, the level that was significantly higher than in WT mice (Figure 9A).

To determine whether IL-17 is required for the high sensitivity of *Pglyrp3* ^−/−^ mice to atopic dermatitis, we compared the severity of ear inflammation in oxazolone-treated *Pglyrp3* ^−/−^ mice in which IL-17 activity was inhibited with neutralizing anti-IL-17 mAb. In vivo neutralization of IL-17 activity in *Pglyrp3* ^−/−^ mice in the oxazolone-induced atopic dermatitis significantly reduced ear inflammation, compared to mice treated with an isotype control IgG (Figure 9B). These results demonstrate that IL-17 is required for full manifestation of severe skin inflammation in *Pglyrp3* ^−/−^ mice in the atopic dermatitis model.

### *Pglyrp3* ^−/−^ and *Pglyrp4* ^−/−^ mice have decreased numbers of Treg cells in the skin

Because WT mice were able to limit skin inflammation in the atopic dermatitis model more effectively than *Pglyrp3* ^−/−^ and *Pglyrp4* ^−/−^ mice, we then tested whether this difference is due to impaired generation or function of regulatory T cells (CD4^+^FoxP3^+^ Treg) in *Pglyrp*-deficient mice.

In the atopic dermatitis model WT mice efficiently recruited Treg cells into the affected skin, as evidenced by an increase in FoxP3-expressing Treg cells in the affected skin shown both by the qRT-PCR (Figure 6A and Figure S1) and by flow cytometry, in which high numbers of CD4^+^FoxP3^+^ Treg cells were found in the affected skin in WT mice (Figure 8B–D). By contrast, atopic dermatitis-sensitive *Pglyrp*-deficient mice (*Pglyrp3* ^−/−^, *Pglyrp4* ^−/−^ and *Pglyrp2* ^−/−^ *Pglyrp3* ^−/−^ mice) all had lower expression of FoxP3 mRNA in the affected ears compared to WT mice (Figure 6A and Figure S1). *Pglyrp3* ^−/−^ mice in the atopic dermatitis model also had significantly lower numbers of CD4^+^FoxP3^+^ Treg cells in the affected skin compared to WT mice measured by flow cytometry (Figure 8B–D). These results suggest impaired recruitment and/or maintenance of Treg cells in the inflamed skin in *Pglyrp3* ^−/−^ and *Pglyrp4* ^−/−^ mice.

To further investigate whether *Pglyrp3* ^−/−^ mice have less efficient generation of induced Treg cells in lymphoid tissues in general or less efficient recruitment and/or maintenance of these cells in the inflamed skin, we compared the numbers of Treg cells in the draining cervical lymph nodes and in the spleen of WT and *Pglyrp3* ^−/−^ mice treated with oxazolone. Oxazolone-treated *Pglyrp3* ^−/−^ mice on day 13 had lower numbers of Treg cells than WT mice in cervical lymph nodes and spleen (Figure 8B). However, at the peak of inflammation (day 20) *Pglyrp3* ^−/−^ mice had similar numbers of Treg cells in the draining lymph nodes and spleen compared to WT mice (Figure 8C). These results indicate that *Pglyrp3* ^−/−^ mice can eventually generate sufficient numbers of induced Treg cells in lymphoid organs and suggest a possible reduced migration and retention of Treg cells in the affected skin.

There could be at least two reasons for this less efficient recruitment of Treg cells to the skin in *Pglyrp3* ^−/−^ mice: insufficient production of Treg-attracting chemokines in the skin, and/or insufficient expression of receptors for these chemokines in Treg cells in *Pglyrp3* ^−/−^ mice. Our results show lower expression of mRNA for Treg-attracting chemokines, CCL-27 (both on days 13 and 20) and CCL-17 (on day 20), in the ears of oxazolone-treated *Pglyrp3* ^−/−^ mice compared to WT mice (Figure 7A and Figures S3 and S5), indicating insufficient production of Treg-attracting chemokines in the skin in *Pglyrp3* ^−/−^ mice. To investigate the second of the above-mentioned possibilities, we determined whether Treg cells in the draining cervical lymph nodes in *Pglyrp3* ^−/−^ mice had sufficient expression of receptors for Treg-attracting chemokines (Ccr4, Ccr8, and Ccr10). The expression of mRNA for Ccr4, Ccr8, and Ccr10 in the draining cervical lymph nodes in oxazolone-treated *Pglyrp3* ^−/−^ mice and WT mice was similar (Figure 8E). These results support the conclusion that Treg cells in the draining lymph nodes in oxazolone-treated *Pglyrp3* ^−/−^ mice have sufficient expression of receptors for Treg-attracting chemokines, but that these Treg cells are not recruited to the inflamed skin, likely because of the insufficient production of Treg-attracting chemokines in the skin (Figure 7A and Figures S3 and S4). Our results thus indicate that Pglyrp3 promotes efficient population of the skin with Treg cells in oxazolone-induced atopic dermatitis.

### Induction of Treg cells in *Pglyrp3* ^−/−^ mice reduces Th17 cells and sensitivity to atopic dermatitis

**Figure 10:**
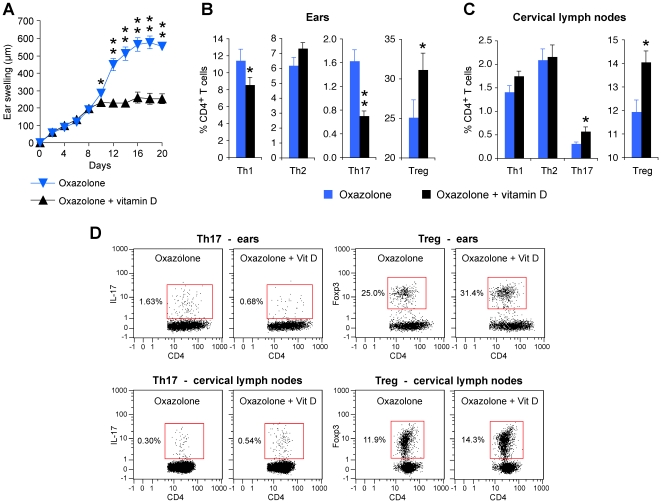
Induction of Treg cells by vitamin D reduces the inflammatory response to oxazolone and decreases the numbers of Th17 cells in the skin of *Pglyrp3* ^−/−^ mice. (**A**) Vitamin D applied to the skin of *Pglyrp3* ^−/−^ mice together with oxazolone reduces ear swelling compared to *Pglyrp3* ^−/−^ mice similarly treated with oxazolone alone. (**B–D**) Vitamin D applied to the skin of *Pglyrp3* ^−/−^ mice together with oxazolone increases the percentages of Treg cells in the ears and cervical lymph nodes and reduces the percentages of Th17 cells in the ears compared to the application of oxazolone alone, measured on day 20 by flow cytometry. Means ± SEM of 8 mice/group (A–C; *, P<0.05; **, P<0.005; oxazolone versus oxazolone + vitamin D) or representative dot plots (D) are shown.

To further investigate the role of Treg cells in high sensitivity of *Pglyrp3* ^−/−^ mice to atopic dermatitis, we induced generation of Treg cells by application of vitamin D to the skin (which is known to induce Treg cells [30], [31]) together with the sensitizing allergen, oxazolone. Vitamin D applied to the ears of *Pglyrp3* ^−/−^ mice together with oxazolone significantly reduced ear swelling compared to *Pglyrp3* ^−/−^ mice similarly treated with oxazolone alone (Figure 10A). Vitamin D applied to the ears (together with oxazolone) also significantly increased the percentages of Treg cells both in the ears and cervical lymph nodes (as expected [30], [31]), and, moreover, it significantly reduced the percentages of Th17 cells in the ears compared to the ears treated with oxazolone alone, measured on day 20 by flow cytometry (Figure 10B–D). Thus, increasing the numbers of Treg cells in the affected skin in *Pglyrp3* ^−/−^ mice to the numbers found in WT mice could revert the inflammatory phenotype of *Pglyrp3* ^−/−^ mice to the less inflammatory phenotype characteristic of WT mice. These results further demonstrate the critical role of Treg cells in preventing high levels of Th17 cells in the skin and excessive inflammation in the oxazolone model of atopic dermatitis. In summary, our results indicate that in WT mice Pglyrp3 and Pglyrp4 promote efficient population of the skin with Treg cells in the experimental model of atopic dermatitis.

## Discussion

Skin diseases such as atopic dermatitis and contact dermatitis involve complex interactions of many cell types. Atopic dermatitis is thought to have Th2 bias [2], [26], but recent findings also show involvement of Th17 cells [32], [33]. The initially identified in vivo role of Th17 cells was promoting some autoimmune diseases and recruitment of PMNs to the sites of inflammation [34]–[37]. However, Th17 cells have many other functions – they play a role in inflammatory bowel diseases, skin diseases, asthma, graft rejection, atherosclerosis, periodontal disease, and arthritis [32], [33], [38]–[40]. We extend these findings by showing that Th17 cells exacerbate skin inflammation in experimental model of atopic dermatitis in a PGRP-dependent manner.

We demonstrate here that *Pglyrp3* ^−/−^ and *Pglyrp4* ^−/−^ mice develop more severe oxazolone-induced atopic dermatitis than WT mice. By contrast, *Pglyrp1* ^−/−^ mice develop less severe oxazolone-induced atopic dermatitis and also less severe contact dermatitis than WT mice. Thus, individual PGRPs play distinct roles in these two models of skin diseases: in WT mice Pglyrp3 and Pglyrp4 protect mice from the development of experimental atopic dermatitis, whereas Pglyrp1 enhances the development of both atopic and contact dermatitis and Pglyrp2 has less effect on both disease models.

The common mechanism underlying these protective effects of PGRPs is decreased recruitment and activity of Treg cells and enhanced production and activation of Th17 cells in the affected skin in *Pglyrp3* ^−/−^ and *Pglyrp4* ^−/−^ mice, which results in more severe inflammation and keratinocyte proliferation. Thus, in WT mice Pglyrp3 and Pglyrp4 promote recruitment and retention of Treg cells in the inflamed skin, which limits over-activation of Th17 cells and protects the skin from exaggerated inflammatory response to allergens.

Our results do show prominent activation of Th2 cells in the oxazolone model of atopic dermatitis and prominent production of IgE, characteristic of Th2 bias in atopic diseases. In our model, however, the main difference at the peak of inflammation between WT and atopic dermatitis-sensitive *Pglyrp*-deficient mice, which determines the enhanced inflammatory responses and higher IgE production in *Pglyrp*-deficient mice, is the overactivation of Th17 cells and reduced numbers of Treg cells in the atopic dermatitis-sensitive mice. Our results indicate that initial Th2 response changes with time to Th17 response and that Th17 cells play an important role in enhancing inflammation and production of IgE in atopic dermatitis. Our results thus further extend recent findings of the enhancing role of Th17 cells in B cell maturation and differentiation [41]–[43]. Therefore, our results indicate that Pglyrp3 and Pglyrp4 are involved in controlling multiple functions of Treg and Th17 cells in the skin in atopic dermatitis.

Contact dermatitis, which is a skin model of type IV hypersensitivity, is usually considered to be mediated by Th1 cells. However, this is an oversimplification, because recent findings show involvement of multiple cell types [2], [4], [5], [44]. Our results show that Pglyrp1 (and other Pglyrps to a lesser extent) promotes Th1 responses, because *Pglyrp1* ^−/−^ mice (and also *Pglyrp2* ^−/−^ and *Pglyrp4* ^−/−^ mice) have reduced inflammation in oxazolone-induced contact dermatitis. This shift of balance to Th1 cells is likely beneficial for the desirable anti-microbial responses and may taper exaggerated inflammatory responses in the skin to allergens. Note that Pglyrp1 is mainly delivered to the sites of inflammation by PMNs, which are usually recruited to fight infections.

Our results show that PGRPs, a family of innate immunity proteins, influence the functions of both innate and adaptive immune cells with an outcome of enhancing the activity of Treg cells and inhibiting the activity of Th17 cells. *Pglyrp3* ^−/−^ and *Pglyrp4* ^−/−^ mice have decreased numbers of Treg cells and increased numbers of Th17 cells in the inflamed skin, compared to WT mice. Allergens and proinflammatory stimuli (such as oxazolone) in WT mice initially induce vigorous cytokine and chemokine production. However, upon chronic exposure, WT mice recruit and maintain large numbers of Treg cells in the inflamed skin and are able to limit the proinflammatory response by reducing the number of proinflammatory genes that are activated and reducing the level of their activation. By contrast, *Pglyrp3* ^−/−^ and *Pglyrp4* ^−/−^ mice have fewer Treg cells and higher numbers of Th17 cells in the affected skin and are unable to limit inflammatory responses. This T cell imbalance in Treg/Th17 cells in *Pglyrp3* ^−/−^ and *Pglyrp4* ^−/−^ mice could come from reduced recruitment of Treg cells and increased recruitment of Th17 cells to the affected skin, and/or from enhanced local differentiation of T cells into Th17 cells (including conversion of Treg cells into Th17 cells) under the influence of locally produced chemokines and cytokines. T cell populations are dynamic and have considerable plasticity based on local cytokine milieu, as, for example, Treg cells can differentiate into Th17 under the influence of locally-produced proinflammatory cytokines [45], [46].

The enhanced recruitment and differentiation of Th17 cells is supported by higher production of Th17 cell promoting cytokines (IL-17, IL-22, IL-23) in the inflamed skin in *Pglyrp3* ^−/−^ and *Pglyrp4* ^−/−^ mice. Decreased recruitment of Treg cells to the inflamed skin is supported by the presence of adequate numbers of Treg cells and adequate expression of receptors for Treg-attracting chemokines on these Treg cells in the draining lymph nodes and spleen in oxazolone-treated *Pglyrp3* ^−/−^ mice, but lower numbers in the skin than in WT mice. These results suggest efficient generation of induced Treg cells in lymphoid organs but defective recruitment to the inflamed skin. This mechanism is further supported by decreased production of Treg cell-attracting chemokines (CCL1, CCL17, CCL27) in the skin of *Pglyrp3* ^−/−^ and *Pglyrp4* ^−/−^ mice. Thus both increased recruitment and generation of Th17 cells and decreased recruitment and retention of Treg cells in the skin are likely responsible for increased inflammation in *Pglyrp3* ^−/−^ and *Pglyrp4* ^−/−^ mice.

Pglyrp3 and Pglyrp4 are primarily expressed in keratinocytes and other epithelial cells, but not in immune cells and stimulation of immune cells does not induce expression of Pglyrp3 and Pglyrp4 [7], [8], [18], [20], [21]. Our results extend these findings by showing expression of Pglyrp3 and Pglyrp4 in differentiated keratinocytes in untreated and oxazolone-treated skin, and no expression of Pglyrp3 and Pglyrp4 in the inflammatory cells infiltrating the skin and in the draining lymph nodes, which primarily contain resting and activated lymphocytes and antigen-presenting cells that migrated from the inflamed skin. Thus, Pglyrp3 and Pglyrp4 most likely exert their anti-inflammatory effect in the skin through their expression in keratinocytes. Keratinocytes are an important local source of chemokines and cytokines, and activation of keratinocytes by proinflammatory stimuli also leads to increased expression of Pglyrp3 and Pglyrp4 in the skin, which correlates with the ability of WT mice to reduce chronic inflammation in the skin. By contrast, increased Pglyrp1 expression in the inflamed skin likely comes from the influx of PMNs, because PMNs express high amounts of Pglyrp1 in their granules and, unlike other PGRPs, Pglyrp1 expression is not increased by proinflammatory stimuli in epithelial cells, including keratinocytes [7], [8], [14], [15]. Thus, the effects of PGRPs in the inflamed skin are likely exerted through a change in the local production of chemokines and cytokines in the skin, which modulates the recruitment and activity of these Treg and Th17 cells. In *Pglyrp3* ^−/−^ and *Pglyrp4* ^−/−^ mice reduced numbers of Treg cells allow dominating expansion of Th17 cells, which can increase inflammatory responses in the atopic dermatitis model, but may reduce Th1-mediated response in the contact dermatitis model by shifting T cell differentiation into Th17 cells instead of Th1 cells. Thus in WT mice, compared to *Pglyrp3* ^−/−^ and *Pglyrp4* ^−/−^ mice, the immune balance is shifted towards Th1 cells, which are protective against microbial infections (rather than Th17 cells) and towards Treg cells that control detrimental inflammation induced by proinflammatory chemicals and allergens. Our results suggest that defects in *Pglyrp3* and *Pglyrp4* genes could be predisposing to atopic dermatitis through the aforementioned shifts in immune homeostasis.

## Materials and Methods

### Ethics statement

All experiments on mice were performed according to the guidelines and approved by the Indiana University School of Medicine–Northwest Institutional Animal Care and Use Committee (approval number IUSM-NW-16).

### Mice

We generated *Pglyrp1* ^−/−^, *Pglyrp2* ^−/−^, *Pglyrp3* ^−/−^, and *Pglyrp4* ^−/−^ mice as described previously [15], [22], [25]. We generated *Pglyrp1* ^−/−^ *Pglyrp2* ^−/−^, *Pglyrp1* ^−/−^ *Pglyrp3* ^−/−^, and *Pglyrp2* ^−/−^ *Pglyrp3* ^−/−^ double knockout mice and *Pglyrp1* ^−/−^ *Pglyrp2* ^−/−^ *Pglyrp3* ^−/−^ and *Pglyrp1* ^−/−^ *Pglyrp2* ^−/−^ *Pglyrp4* ^−/−^ triple knockout mice by breeding single and double knockout mice (all on BALB/c background) and screening for homozygous deletion of each *Pglyrp* gene by PCR analysis of genomic DNA as previously described [15], [22], [25]. The lack of expression of the *Pglyrp* genes was confirmed by qRT-PCR in mRNA from the ears. Double and triple homozygous *Pglyrp* knockout mice were viable and fertile, bred normally, and yielded the expected male∶female ratios and similar litter size as the wild type and heterozygous mice. They had similar weight as the WT and single *Pglyrp* knockout mice and developed normally with no obvious defects. Their major internal organs had normal macroscopic appearance, and normal histological appearance on hematoxylin/eosin-stained sections.

All mice used in experiments were 8–10 week-old and on BALB/c background. The original colony founder WT BALB/c breeder mice were obtained from Harlan-Sprague-Dawley. All knockout mice were backcrossed to the same WT BALB/c mice from our breeding colony, and all WT and knockout mice were bred and kept under conventional pathogen-free conditions in the same room in our facility to minimize the influence of differences in the environment. For each experiment, mice from several different cages and breeder pairs were used. The BALB/c background of *Pglyrp*-deficient mice and their negative status for all common viral and bacterial pathogens and parasites were confirmed as previously described [22].

### Oxazolone atopic dermatitis and contact dermatitis models

To induce atopic dermatitis female mice were first sensitized with 10 µl of 5% oxazolone (in 80% acetone, 20% olive oil) applied to the abdomen (after removing hair with Nair cream); 6 days later applications of 30 µl of 0.1% oxazolone (in 80% acetone, 20% olive oil) to each ear (15 µl to each side) were started (day 0) and continued every other day through day 18 [26]. In some experiments to induce Treg cells [30], [31] 3 µM vitamin D (1α,25-dihydroxyvitamin D_3_ from Sigma) was added to the oxazolone solution and used for the initial sensitization and applications to the ears. Ear thickness was measured each time before oxazolone application with Digimatic Micrometer (Mitutoyo, Japan) under constant pressure at the lowest setting. Ear swelling was determined by subtracting the untreated ear thickness. The significance of differences in ear swelling was determined using *t*-test.

To induce contact dermatitis female mice were first sensitized with 50 µl of 2% oxazolone (Sigma, in 80% acetone, 20% olive oil) applied to the abdomen (after removing hair with Nair cream) and 5 µl applied to each paw. The contact dermatitis reaction was then elicited 6 days later with a single application of 20 µl of 1% oxazolone (in 80% acetone, 20% olive oil) to each ear (10 µl to each side) [27], [28]. Ear thickness was measured as described above before and 24 and 48 hrs after oxazolone application, and ear swelling was determined by subtracting the untreated ear thickness. The significance of differences in ear swelling was determined using *t*-test.

### Histology and immunohistochemistry

For histological analysis ears were fixed in Bouin's fixative, postfixed in 70% ethanol, and embedded in paraffin, and 5 µm cross-sections were stained with hematoxylin/eosin, and evaluated microscopically. For immunohistochemistry, antibodies to mouse Pglyrp3 and Pglyrp4 were obtained by immunizing rabbits with peptides corresponding to the following amino acids: CLVPQHSEIPKKA for Pglyrp3 (exon 5), and CWENPQTDQVSEG for Pglyrp4 (exon 2), coupled to KLH, followed by affinity purification on SulfoLink gel (Pierce) with corresponding peptides linked through the N-terminal Cys, elution with Tris-glycine buffer, pH 2.5, and dialysis against PBS, pH 7.2. A rabbit IgG antibody to a different peptide, which did not react with mouse Pglyrp3 and Pglyrp4, prepared and purified by the same method, was used as a negative control. Paraffin 5 µm cross-sections of were stained by the immunoperoxidase method as previously described [8], including standard deparaffinization, re-hydration, quenching of endogenous peroxidase by 30 min incubation in 0.3% H_2_O_2_, and incubation with 0.5 µg/ml of anti-Pglyrp3 or Pglyrp4 antibodies or control IgG overnight, followed by biotinylated second Ab and Vectastain Elite ABC kit (Vector) with DAB as a substrate (which generates brown reaction product) and counterstaining with hematoxylin (blue).

### RNA and quantitative real-time reverse transcription PCR (qRT-PCR)

RNA was isolated from either the entire untreated or treated ears or lymph nodes using the TRIZOL method (InVitrogen), followed by digestion with RNase-free DNase (Qiagen) and purification on RNeasy spin columns using RNeasy Minikit (Qiagen). Quantitative reverse transcription real-time PCR (qRT-PCR) was used to quantify the amounts of mRNA in the ears or lymph nodes using custom RT^2^ Profiler PCR Arrays designed by us and manufactured by Qiagen/SA Biosciences, as previously described [22], [25]. The arrays typically included 30 to 44 assay genes, 5 housekeeping genes and reverse transcription efficiency and DNA contamination controls. All primer sets were from Qiagen/SA Biosciences, except the following primers designed by us: Pglyrp1, exons 1 and 2 primers, GTGGTGATCTCACACACAGC and GTGTGGTCACCCTTGATGTT; Pglyrp2, exons 3 and 4 primers, ACCAGGATGTGCGCAAGTGGGAT and AGTGACCCAGTGTAGTTGCCCA; and Pglyrp4, exons 4 and 5 primers, CGACCAGGGCTACAAGAA and CCAGGCAGTCTTCACTTTTC. cDNA was synthesized from 2 µg of RNA using RT^2^ PCR Array First Strand Kit (Qiagen/SA Biosciences) and the arrays were performed according to the manufacturer instructions using Qiagen/SA Biosciences Master Mix. The lists of genes are provided in the figures. The experiments were performed on RNA pooled from 4–5 mice/group and repeated 3 times usually with another set of 4–5 mice/group (usually total of 8–10 mice per treatment).

For each gene, ΔCt was calculated using the same threshold (0.2) for all genes and Ct≤35 considered as no expression, followed by normalization to 5 housekeeping genes (Hsp90ab1, Gusb, Hprt1, Gapdh, and Actb) included in each array, followed by calculation of ΔΔCt for each gene from two arrays: ΔΔCt  =  ΔCt1−ΔCt2, where ΔCt1 is the oxazolone treated mice and ΔCT2 is the untreated mice, using the program provided by Qiagen/SA Biosciences. This calculation gives the fold increase in expression of each gene in the treated mice versus untreated mice per µg RNA. The genomic DNA contamination controls, reverse transcription controls, and positive PCR controls were included in each array and were all passed. Additional control to assure amplification from RNA, but not from possible contaminating DNA included parallel reaction sets from which reverse transcriptase was omitted, and which showed no amplification. To compare baseline gene expression in untreated mice, ΔCT1 was from untreated PGRP-deficient mice and ΔCT2 was from untreated WT mice.

The results were reported as mean fold increases after oxazolone treatment (treated/untreated) for WT mice, or ratios of fold increases in *Pglyrp*-deficient to WT mice, calculated as follows: [(*Pglyrp* ^−/−^ treated)/(*Pglyrp* ^−/−^ untreated)]/[(WT treated)/(WT untreated)] and presented as heat maps or bar graphs. The latter fold differences (ratios) of >1 or <1 reflect higher or lower expression levels of the genes (respectively) in *Pglyrp*-deficient than in WT mice. Heat maps were generated using Java TreeView after converting <1 ratios to negative fold difference using the formula: (−1)/ratio. In some bar graph figures, <1 ratios were also converted to negative fold difference using the formula: (−1)/ratio. The significance of differences in gene activation between groups of mice was determined using the two-sample one-tailed *t*-test, and typically the differences of >2 fold were significant at P<0.05.

Expression of mRNA for PGRPs and some chemokine receptors was similarly measured by qRT-PCR using Qiagen/SA Biosciences First Strand Kit (with random primers) and amplification for 40 cycles with Qiagen/SA Biosciences SYBR Green Master Mix, and calculated using comparative cycle threshold method with 5 housekeeping genes (Hsp90ab1, Gusb, Hprt1, Gapdh, and Actb) as controls.

### Isolation of cells and flow cytometry

Mouse ears were placed in RPMI-1640 with 3 mg/ml of Dispase II (Roche), separated into dorsal and ventral halves and scored on the dermal side with a scalpel. The tissue was digested for 8 hrs at 37°C in 5% CO_2_. Dermis was then separated from the epidermis and epidermis was further digested with 0.25% trypsin in RPMI-1640 for 10 min at 37°C. Cells were washed twice with RPMI-1640 with 5% fetal bovine serum (FBS) and incubated for 20 hrs in the same medium at 37°C in 5% CO_2_. Cells were then strained through a 40 µm filter and resuspended at 2.0×10^7^ cells/ml in RPMI-1640 with 5% FBS. Single cells from cervical lymph nodes and spleen were obtained by passing the tissue through a 40 µm filter, red blood cells were removed from the spleen cells with a lysis buffer (Biolegend), and cells were suspended at 2.0×10^7^ cells/ml in RPMI-1640 with 5% FBS.

1×10^6^ cells were stained with CD4-APC (clone RM4-5, Biolegend) antibody for 20 min at 4°C. CD4-stained cells were then stained for Foxp3-PE (clone FJK-16s, eBioscience) or for cytokines IFN-γ-PE (clone XMG1.2), IL4-PE (clone 11B11) and IL-17-PE (clone TC11-18H10.1) with antibodies from Biolegend, used at 0.2 mg/1×10^6^ cells according to Biolegend protocols using Biolegend buffers. Prior to staining for cytokines, CD4-APC stained cells were activated with TPA (12-O-Tetradecanoylphorbol 13-acetate, 25 ng/ml) and ionomycin (250 ng/ml) in the presence of the Golgi inhibitor, monensin, for 4 hrs at 37°C in 5% CO_2_. Cells were analyzed by flow cytometry using MACSQuant (Miltenyi) cytometer. Foxp3, IFN-γ, IL-4 and IL-17 positive cells were measured within the CD4^+^ gate.

### Neutralization of IL-17 and ELISA

IL-17 was neutralized by intravenous injections of anti-IL-17 mAb (specific for IL-17A and not reactive with IL-1F, rat clone 50104, endotoxin-free from R&D Systems) 100 µg at sensitization and 50 µg on days 0, 3, 6, 9, 12, and 15 of oxazolone treatment. Control mice were similarly treated with isotype control rat IgG2aκ mAb (clone 16-4321, endotoxin-free from eBioscience). The amount of CXCL-1 in ears was measured by ELISA as previously described [25], after each ear was homogenized with Polytron in 0.5 ml of PBS (without Ca/Mg) with 1 mM EDTA, 1 mM PMSF, 1∶100 dilution of protease inhibitors (Sigma P1860), and 0.5% Triton X-100, followed by sonication and centrifugation.

## Supporting Information

Figure S1 ***Pglyrp3*** **^−/−^ and** ***Pglyrp4*** **^−/−^ mice have increased Th17 cells in the affected skin in the oxazolone atopic dermatitis model.** Expression of a panel of marker genes characteristic of various inflammatory cell types in the ears of mice after sensitization and 10 applications of oxazolone to the ears every other day measured by qRT-PCR (day 20) is shown. For WT mice (top panel), the ratio of the amount of mRNA in oxazolone-treated to untreated mice for each gene (fold induction by oxazolone) is shown; for *Pglyrp* ^−/−^ mice, the results are the ratios of fold induction of each gene by oxazolone in *Pglyrp* ^−/−^ mice to fold induction of each gene by oxazolone in WT mice (which represents the fold difference in the response to oxazolone in *Pglyrp* ^−/−^ versus WT mice). The results are means ± SEM of 3 arrays from 4–5 mice/group and are shown as heat maps in Figure 6A in the main article.
(PDF)

Figure S2 **In the oxazolone contact dermatitis model in** ***Pglyrp1*** **^−/−^ and** ***Pglyrp2*** **^−/−^ mice most cell types are decreased in the affected skin, but Treg cells and B cells are increased.** Expression of a panel of marker genes characteristic of various inflammatory cell types in the ears of mice after sensitization and 6 hrs after single application of oxazolone to the ears measured by qRT-PCR is shown. For WT mice (top panel), the ratio of the amount of mRNA in oxazolone-treated to untreated mice for each gene (fold induction by oxazolone) is shown; for *Pglyrp* ^−/−^ mice, the results are the ratios of fold induction of each gene by oxazolone in *Pglyrp* ^−/−^ mice to fold induction of each gene by oxazolone in WT mice (which represents the fold difference in the response to oxazolone in *Pglyrp* ^−/−^ versus WT mice). The results are means ± SEM of 3 arrays from 4–5 mice/group and are shown as heat maps in Figure 6B in the main article.
(PDF)

Figure S3 **Multiple inflammatory and immune genes are induced at an early stage of oxazolone model of atopic dermatitis.** Expression of a panel of cytokines, chemokines, and other marker genes characteristic of Th1, Th2, Th17, Treg, NK, and other cell types in the ears of mice after sensitization and 7 applications of oxazolone to the ears every other day measured by qRT-PCR (day 13). For WT mice (top panel), the ratio of the amount of mRNA in oxazolone-treated to untreated mice for each gene (fold induction by oxazolone) is shown; for *Pglyrp3* ^−/−^ or *Pglyrp4* ^−/−^ mice, the results are the ratios of fold induction of each gene by oxazolone in *Pglyrp* ^−/−^ mice to fold induction of each gene by oxazolone in WT mice (which represents the fold difference in the response to oxazolone in *Pglyrp* ^−/−^ versus WT mice). The results are means ± SEM of 3 arrays from 4–5 mice/group and are shown as heat maps in Figure 7A in the main article.
(PDF)

Figure S4 **Th17 gene expression profile is preferentially induced in the oxazolone model of atopic dermatitis in** ***Pglyrp3*** **^−/−^ and** ***Pglyrp4*** **^−/−^ mice.** Expression of a panel of cytokines, chemokines, and other marker genes characteristic of Th1, Th2, Th17, Treg, NK, and other cell types in the ears of mice after sensitization and 10 applications of oxazolone to the ears every other day shows higher induction of several Th17 marker genes in *Pglyrp3* ^−/−^ and *Pglyrp4* ^−/−^ compared to WT mice measured by qRT-PCR. For WT mice (top panel), the ratio of the amount of mRNA in oxazolone-treated to untreated mice for each gene (fold induction by oxazolone) is shown; for *Pglyrp* ^−/−^ mice, the results are the ratios of fold induction of each gene by oxazolone in *Pglyrp* ^−/−^ mice to fold induction of each gene by oxazolone in WT mice (which represents the fold difference in the response to oxazolone in *Pglyrp* ^−/−^ versus WT mice). The results are means ± SEM of 3–4 arrays from 4–5 mice/group and are shown as heat maps in Figure 7A in the main article.
(PDF)

Figure S5 **Expression of most immune genes is reduced in the oxazolone model of contact dermatitis in** ***Pglyrp*** **^−/−^mice.** Expression of a panel of cytokines, chemokines, and other marker genes characteristic of Th1, Th2, Th17, Treg, NK, and other cell types in the ears of mice after sensitization and a single application of oxazolone to the ears shows lower induction of immune marker genes in *Pglyrp* ^−/−^ mice compared to WT mice measured by qRT-PCR. For WT mice (top panel), the ratio of the amount of mRNA in oxazolone-treated to untreated mice for each gene (fold induction by oxazolone) is shown; for *Pglyrp* ^−/−^ mice, the results are the ratios of fold induction of each gene by oxazolone in *Pglyrp* ^−/−^ mice to fold induction of each gene by oxazolone in WT mice (which represents the fold difference in the response to oxazolone in *Pglyrp* ^−/−^ versus WT mice, negative numbers show lower gene induction in *Pglyrp* ^−/−^ than in WT mice). The results are means ± SEM of 3–4 arrays from 4–5 mice/group and are shown as heat maps in Figure 7B in the main article.
(PDF)
