# Supplementary figures and images for: Differential Effects of Peptidoglycan Recognition Proteins on Experimental Atopic and Contact Dermatitis Mediated by Treg and Th17 Cells
Source: PLoS One. 2011 Sep 16;6(9):e24961. doi: 10.1371/journal.pone.0024961 (PMC3174980; doi:10.1371/journal.pone.0024961)

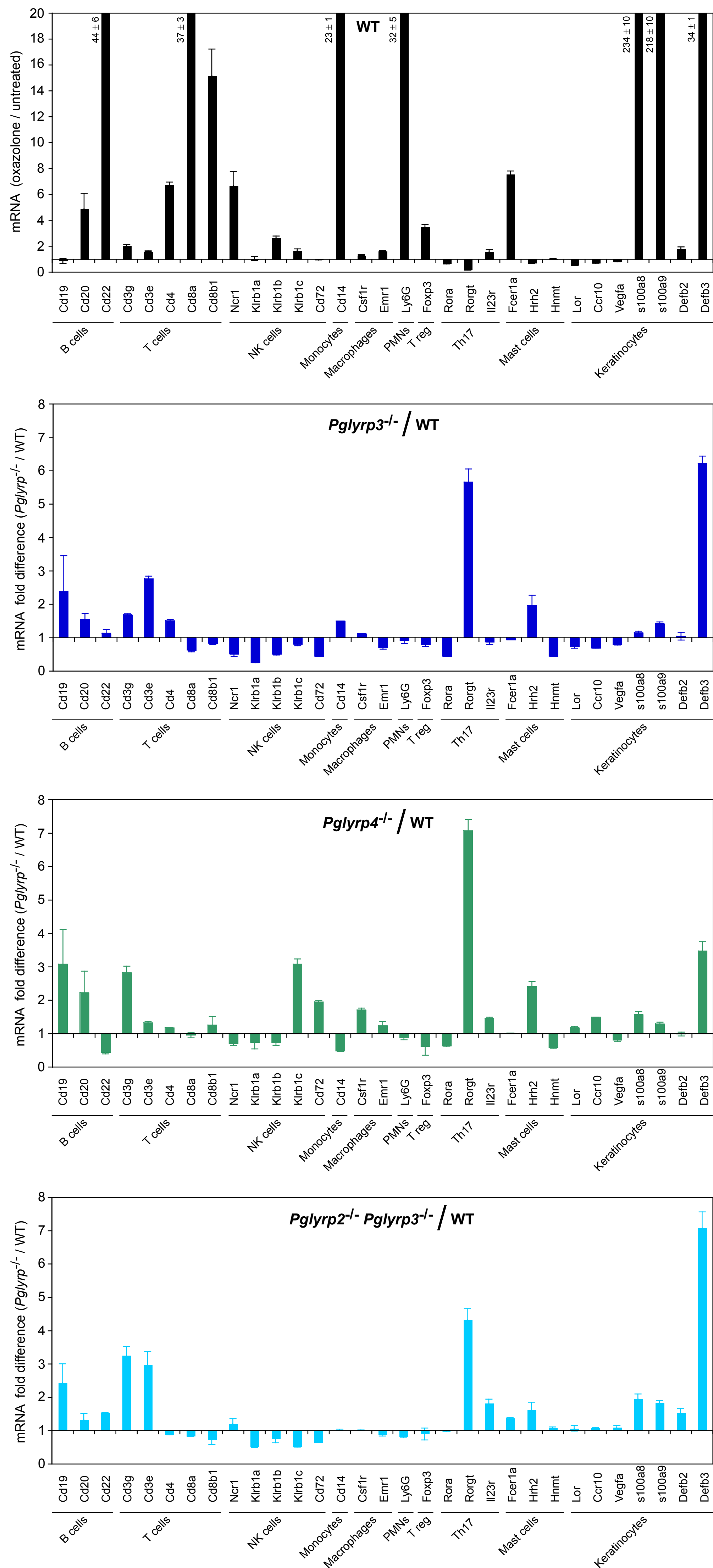

Figure S1

Supplement: Figure S1 — Pglyrp3 −/− and Pglyrp4 −/− mice have increased Th17 cells in the affected skin in the oxazolone atopic dermatitis model. Expression of a panel of marker genes characteristic of various inflammatory cell types in the ears of mice after sensitization and 10 applications of oxazolone to the ears every other day measured by qRT-PCR (day 20) is shown. For WT mice (top panel), the ratio of the amount of mRNA in oxazolone-treated to untreated mice for each gene (fold induction by oxazolone) is shown; for Pglyrp −/− mice, the results are the ratios of fold induction of each gene by oxazolone in Pglyrp −/− mice to fold induction of each gene by oxazolone in WT mice (which represents the fold difference in the response to oxazolone in Pglyrp −/− versus WT mice). The results are means ± SEM of 3 arrays from 4–5 mice/group and are shown as heat maps in Figure 6A in the main article. (PDF) [file pone.0024961.s001.pdf]

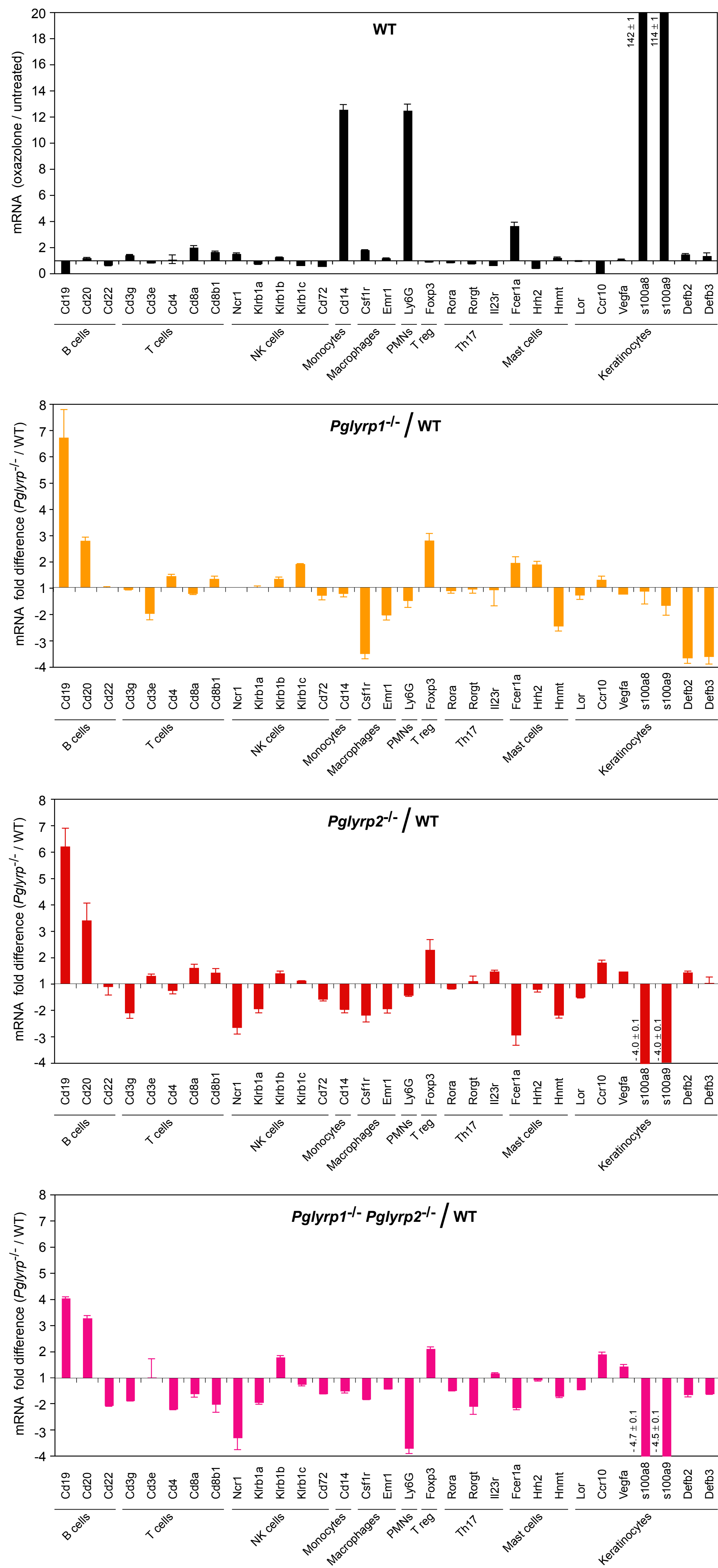

Figure S2

Supplement: Figure S2 — In the oxazolone contact dermatitis model in Pglyrp1 −/− and Pglyrp2 −/− mice most cell types are decreased in the affected skin, but Treg cells and B cells are increased. Expression of a panel of marker genes characteristic of various inflammatory cell types in the ears of mice after sensitization and 6 hrs after single application of oxazolone to the ears measured by qRT-PCR is shown. For WT mice (top panel), the ratio of the amount of mRNA in oxazolone-treated to untreated mice for each gene (fold induction by oxazolone) is shown; for Pglyrp −/− mice, the results are the ratios of fold induction of each gene by oxazolone in Pglyrp −/− mice to fold induction of each gene by oxazolone in WT mice (which represents the fold difference in the response to oxazolone in Pglyrp −/− versus WT mice). The results are means ± SEM of 3 arrays from 4–5 mice/group and are shown as heat maps in Figure 6B in the main article. (PDF) [file pone.0024961.s002.pdf]

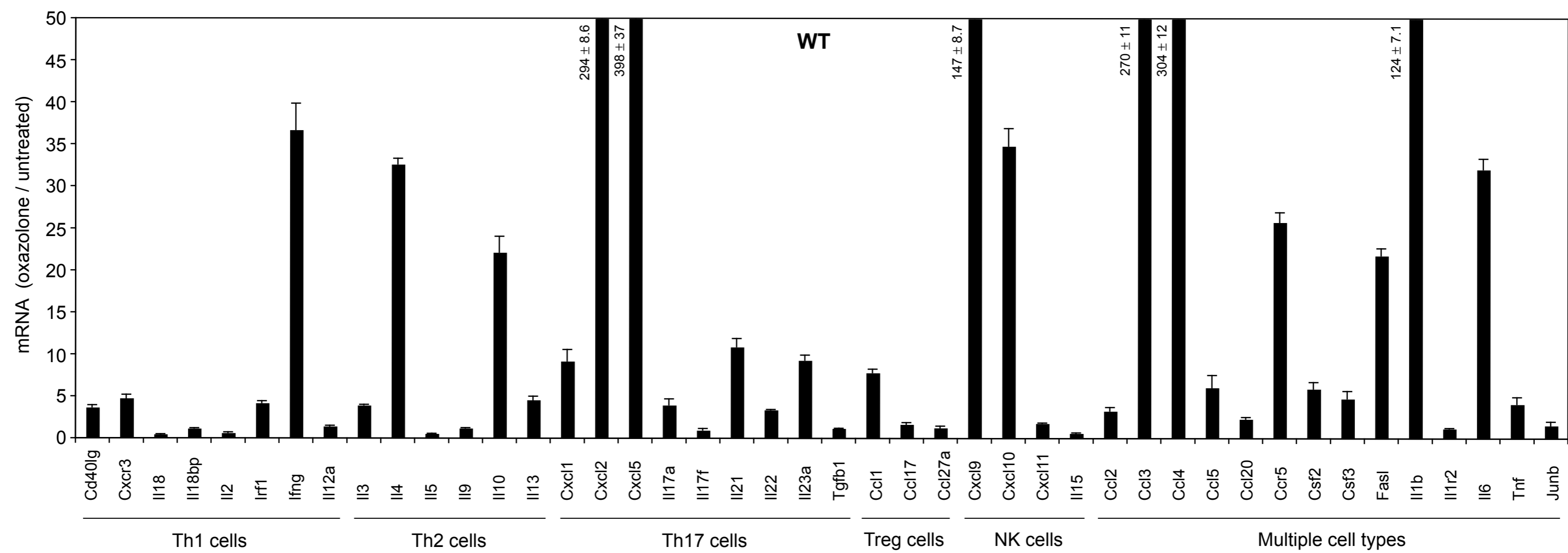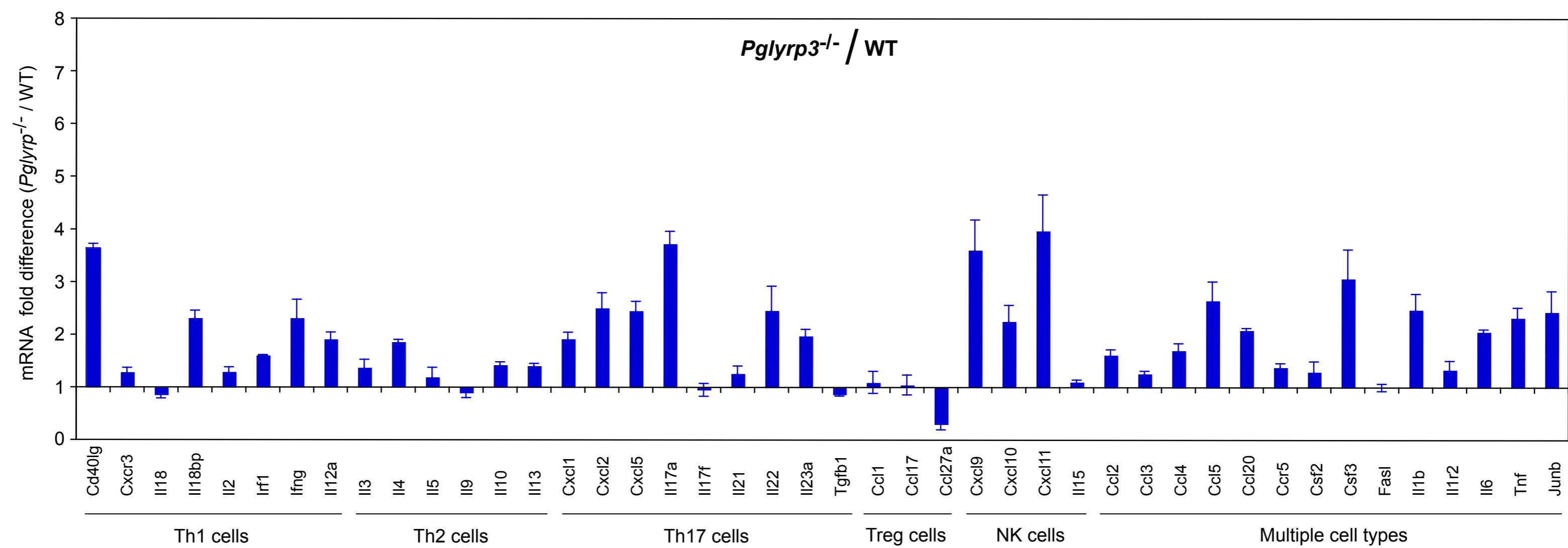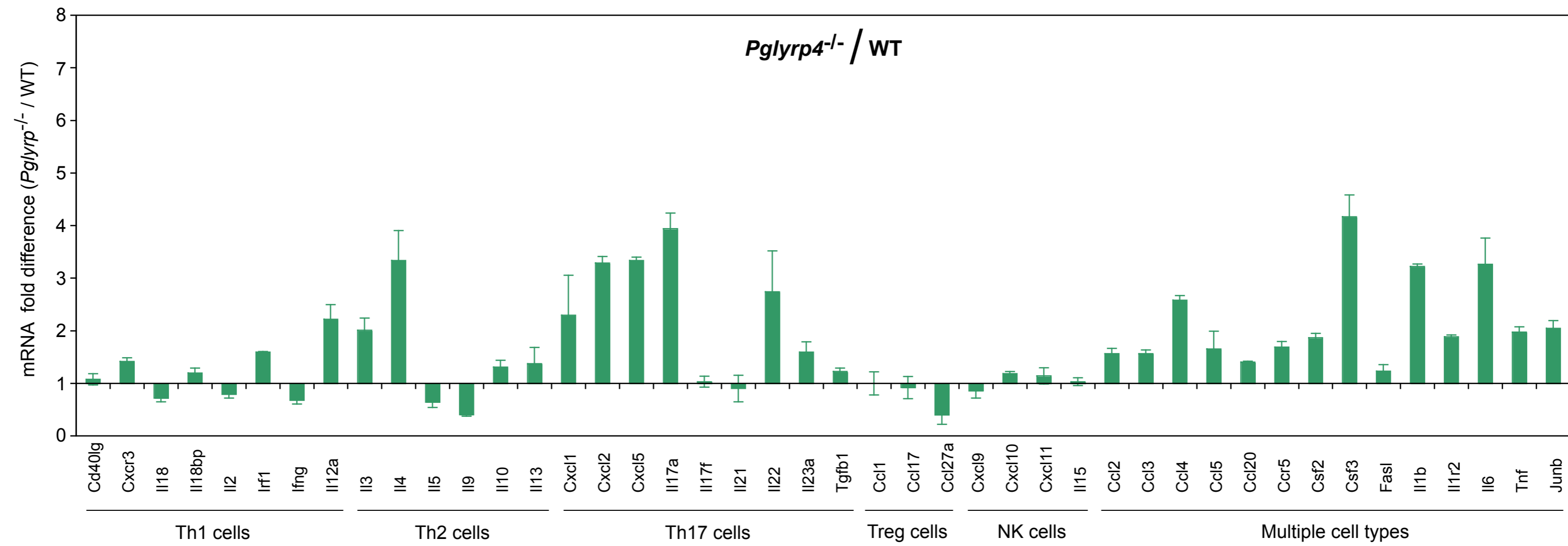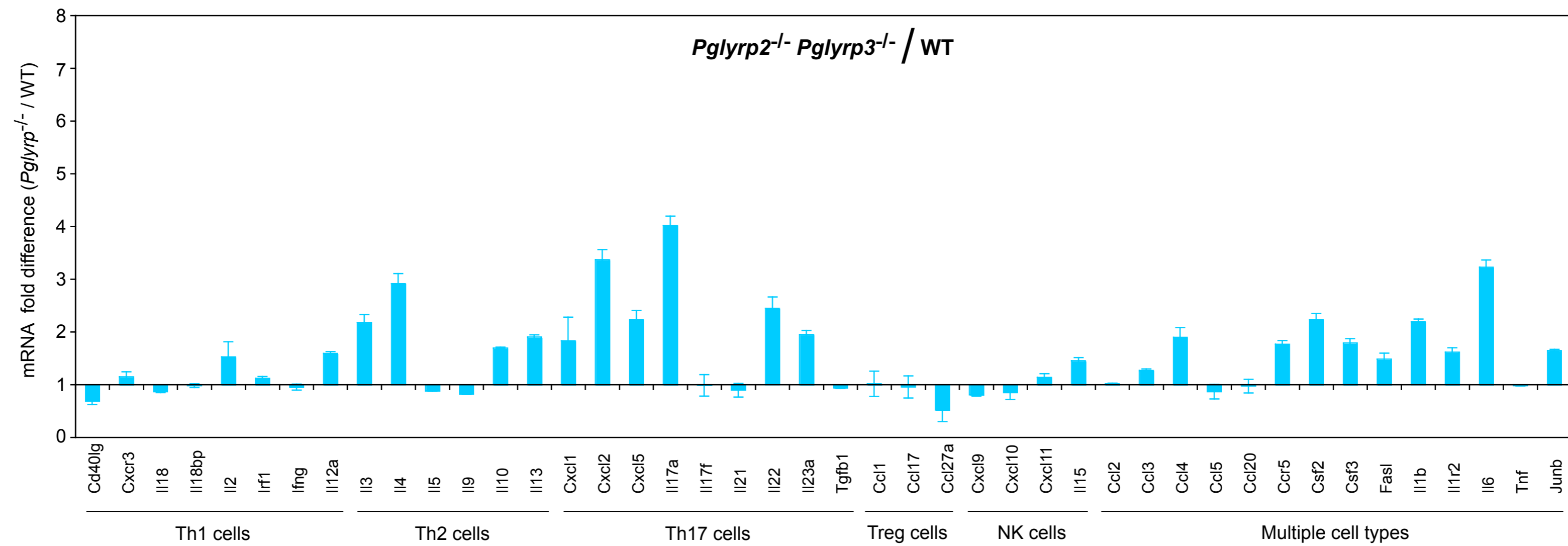

Figure S3

Supplement: Figure S3 — Multiple inflammatory and immune genes are induced at an early stage of oxazolone model of atopic dermatitis. Expression of a panel of cytokines, chemokines, and other marker genes characteristic of Th1, Th2, Th17, Treg, NK, and other cell types in the ears of mice after sensitization and 7 applications of oxazolone to the ears every other day measured by qRT-PCR (day 13). For WT mice (top panel), the ratio of the amount of mRNA in oxazolone-treated to untreated mice for each gene (fold induction by oxazolone) is shown; for Pglyrp3 −/− or Pglyrp4 −/− mice, the results are the ratios of fold induction of each gene by oxazolone in Pglyrp −/− mice to fold induction of each gene by oxazolone in WT mice (which represents the fold difference in the response to oxazolone in Pglyrp −/− versus WT mice). The results are means ± SEM of 3 arrays from 4–5 mice/group and are shown as heat maps in Figure 7A in the main article. (PDF) [file pone.0024961.s003.pdf]

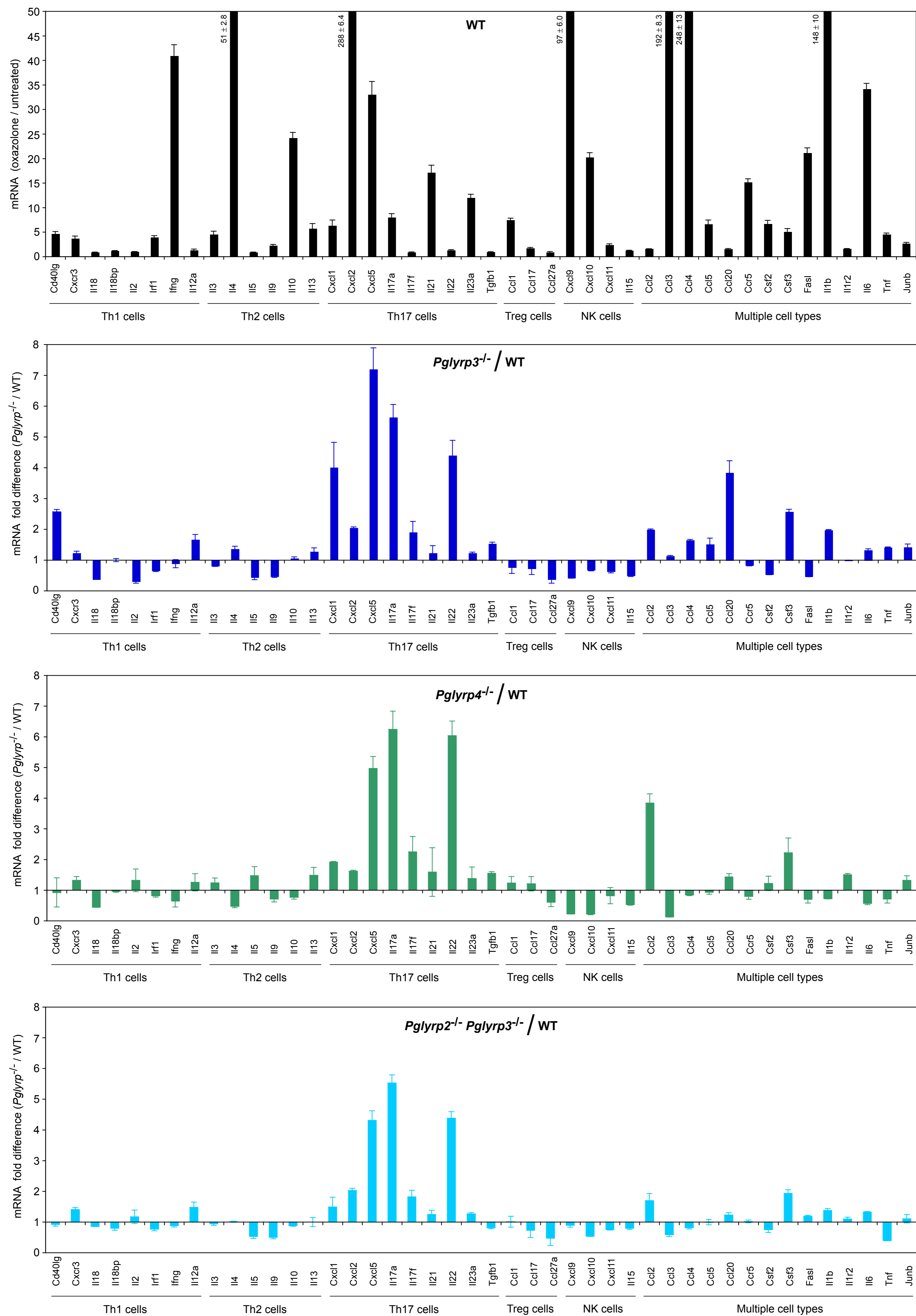

Figure S4

Supplement: Figure S4 — Th17 gene expression profile is preferentially induced in the oxazolone model of atopic dermatitis in Pglyrp3 −/− and Pglyrp4 −/− mice. Expression of a panel of cytokines, chemokines, and other marker genes characteristic of Th1, Th2, Th17, Treg, NK, and other cell types in the ears of mice after sensitization and 10 applications of oxazolone to the ears every other day shows higher induction of several Th17 marker genes in Pglyrp3 −/− and Pglyrp4 −/− compared to WT mice measured by qRT-PCR. For WT mice (top panel), the ratio of the amount of mRNA in oxazolone-treated to untreated mice for each gene (fold induction by oxazolone) is shown; for Pglyrp −/− mice, the results are the ratios of fold induction of each gene by oxazolone in Pglyrp −/− mice to fold induction of each gene by oxazolone in WT mice (which represents the fold difference in the response to oxazolone in Pglyrp −/− versus WT mice). The results are means ± SEM of 3–4 arrays from 4–5 mice/group and are shown as heat maps in Figure 7A in the main article. (PDF) [file pone.0024961.s004.pdf]

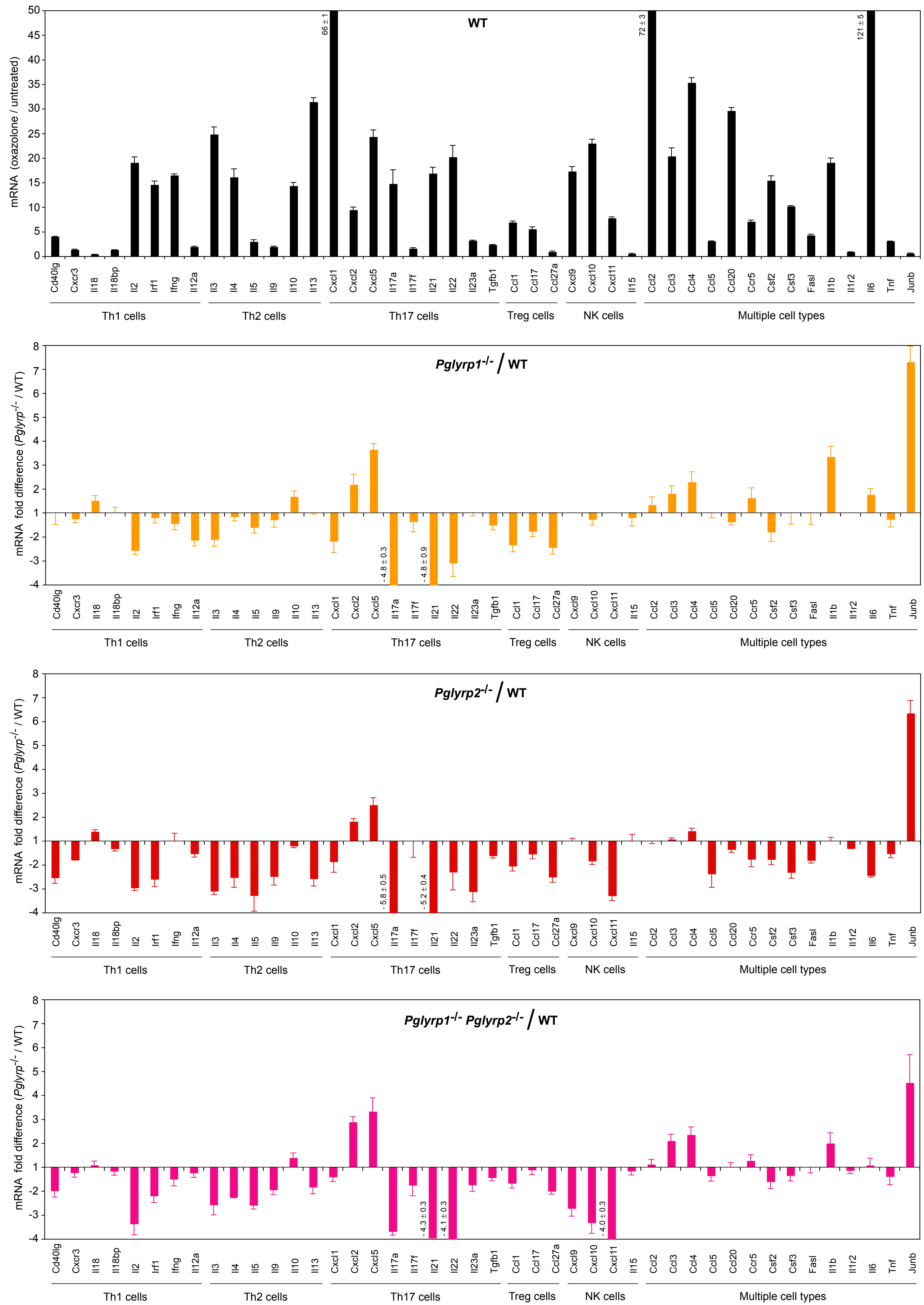

Figure S5

Supplement: Figure S5 — Expression of most immune genes is reduced in the oxazolone model of contact dermatitis in Pglyrp −/−mice. Expression of a panel of cytokines, chemokines, and other marker genes characteristic of Th1, Th2, Th17, Treg, NK, and other cell types in the ears of mice after sensitization and a single application of oxazolone to the ears shows lower induction of immune marker genes in Pglyrp −/− mice compared to WT mice measured by qRT-PCR. For WT mice (top panel), the ratio of the amount of mRNA in oxazolone-treated to untreated mice for each gene (fold induction by oxazolone) is shown; for Pglyrp −/− mice, the results are the ratios of fold induction of each gene by oxazolone in Pglyrp −/− mice to fold induction of each gene by oxazolone in WT mice (which represents the fold difference in the response to oxazolone in Pglyrp −/− versus WT mice, negative numbers show lower gene induction in Pglyrp −/− than in WT mice). The results are means ± SEM of 3–4 arrays from 4–5 mice/group and are shown as heat maps in Figure 7B in the main article. (PDF) [file pone.0024961.s005.pdf]
